# TkSRPP3/4 interactors TkGGR1 and TkLIL3 link plastid-like organelles with isoprenoid metabolism in *Taraxacum koksaghyz* latex

**DOI:** 10.1007/s00299-025-03537-3

**Published:** 2025-06-24

**Authors:** Silva Melissa Wolters, Lukas Schwarz, Ronja Khairat, Kristina Sturm, Boje Müller, Nicole van Deenen, Richard M. Twyman, Dirk Prüfer, Christian Schulze Gronover

**Affiliations:** 1 Fraunhofer Institute for Molecular Biology and Applied Ecology IME, Münster, Germany; 2 Institute of Plant Biology and Biotechnology, University of Münster, Münster, Germany; 3 TRM Ltd, Scarborough, UK

**Keywords:** Small rubber particle proteins, Geranylgeranyl reductase, LIL3, Latex, Frey–Wyssling particles, Geranylgeranyl diphosphate synthase

## Abstract

**Key message:**

The presence of plastid-like structures in the latex of the Russian dandelion *Taraxacum koksaghyz* and interactions involving plastid-associated TkGGR1 with TkSRPP3, TkGGPS6 and TkLIL3 may confer TkSRPP-mediated stress tolerance.

**Abstract:**

The latex of the Russian dandelion *Taraxacum koksaghyz* is a rich source of natural rubber (NR) but other facets of its metabolism and physiology have been largely neglected. Small rubber particle proteins (SRPPs) contribute to NR biosynthesis by stabilizing rubber particles and are also linked to stress responses. The identification of geranylgeranyl reductase (GGR1) as potential interactor of TkSRPP3 in our previous study prompted its detailed investigation because GGRs normally reduce geranylgeranyl groups to phytol or phytyl diphosphate for chlorophyll synthesis in chloroplasts. Here we determined the latex-specific expression and phytol-producing activity of GGR1, and confirmed its interaction with TkSRPP3. Metabolic analysis of plants with altered *TkGGR1* expression levels in latex revealed its involvement in tocopherol but not NR synthesis in roots, whereas a second, leaf-specific GGR was responsible for chlorophyll synthesis. We found that a geranylgeranyl diphosphate synthase (GGPS) and light-harvesting-like 3 protein (LIL3) were co-expressed in latex and translocated into *Nicotiana benthamiana* chloroplasts, as we also observed for TkGGR1. We confirmed that TkGGR1 interacted with TkGGPS6 and TkLIL3 inside chloroplasts and detected an extraplastidial interaction between TkLIL3 and TkSRPP4. In situ analysis of mVenus-tagged TkGGR1 indicated its localization in plastid-like structures in *T.* *koksaghyz* latex, which lacks conventional chloroplasts. We therefore hypothesized the presence of a TkGGR1-containing multiprotein complex within Frey–Wyssling-like particles in latex that may confer oxidative stress tolerance. This study provides insight into a previously undescribed branch of isoprenoid metabolism and cellular biology of NR-producing laticifers in *T.* *koksaghyz*.

**Supplementary Information:**

The online version contains supplementary material available at 10.1007/s00299-025-03537-3.

## Introduction

The latex of the Russian dandelion *Taraxacum koksaghyz* provides an abundant source of high-quality natural rubber (NR). The biosynthesis of this valuable biopolymer has been studied in detail, whereas other aspects of latex metabolism and physiology have received much less attention. Small rubber particle proteins (SRPPs) 3, 4 and 5 are highly abundant in latex, particularly on the surface of organelles known as rubber particles, where they facilitate NR biosynthesis (Collins-Silva et al. 2012; Hillebrand et al. 2012). *Taraxacum* SRPPs 3/4/5 are also involved in stress responses, which was demonstrated by enhanced drought stress tolerance upon overexpression in transgenic plants and their stress-responsive transcription (H. He et al. 2024; Laibach et al. 2018; Wu et al. 2024). This is in line with the physiological role of latex in defense and stress responses (Böttner et al. 2023; Huber et al. 2016; Konno 2011; Salomé Abarca et al. 2019) and is an rare example of research targeting the molecular biology of latex aside from NR synthesis.

It is currently unclear how exactly SRPPs promote NR biosynthesis. Knockdown experiments indicated a role in rubber particle stability and dispersity (Collins-Silva et al. 2012; Hillebrand et al. 2012), and a recent protein interaction screen for TkSRPPs 3/4/5 suggested that TkSRPPs engage with other rubber particle proteins, including the *cis*-prenyltransferase (*cis*PT) complex that catalyzes NR (poly(*cis*−1,4-isoprene)) polymerization (Wolters et al. 2024). The TkSRPP3/4/5 interactomes also revealed that TkSRPPs are connected to other branches of the rich isoprenoid network in latex (for an overview of the isoprenoid metabolic network see Supplementary Fig. 9). Understanding the interplay between these branches and how they are regulated will provide insight into NR biosynthesis as a component of wider latex physiology.

One candidate TkSRPP3 interactor caught our particular attention because it was homologous to geranylgeranyl reductases (GGRs), which are mostly known to facilitate chlorophyll and tocopherol synthesis in chloroplasts. However, latex lacks chlorophyll and does not contain genuine chloroplasts (Liu et al. 2015; Tanaka et al. 1999). GGR catalyzes the reduction of geranylgeranyl diphosphate (GGPP) to phytyl diphosphate (PhyPP), both of which are found as chlorophyll side chains, and PhyPP is also required for the synthesis of antioxidant tocopherols. The reduction of GGPP via dihydro- (DHGG) and tetrahydro- (THGG) derivatives consumes three NADPH molecules. It is not yet clear whether GGR can use both esterified and free geranylgeraniol (GGOH) or GGPP as substrates (Hirose et al. 2022; Keller et al. 1998; Soll & Schultz 1981; Tanaka et al. 2010). GGPP is an unsaturated prenyl chain assembled from four C_5_ isoprene units. This monomeric isopentenyl diphosphate (IPP) unit and its allylic isomer dimethylallyl diphosphate (DMAPP) are produced by the cytoplasmic mevalonate (MVA) and plastidial methylerythritol (MEP) pathways. A geranyl diphosphate synthase (GPS) transfers one DMAPP to IPP forming geranyl diphosphate (GPP), and this can be further elongated by one or two IPP units to produce farnesyl diphosphate (FPP) and GGPP, catalyzed by the *trans*-prenyltransferases (TPTs) farnesyl diphosphate synthase (FPS) and geranylgeranyl diphosphate synthase (GGPS), respectively (Tholl 2015). However, TPT substrate and product specificities are not strict and can be modulated by heterodimerization (Conart et al. 2023; Orlova et al. 2009; Takaya et al. 2003; Wang and Dixon 2009). GGPS can be found in the cytosol, mitochondria and plastids (Beck et al. 2013; Ruiz-Sola et al. 2016a, b), and in the latter they provide GGPP for the synthesis of photosynthesis-related isoprenoids including photosynthetic pigments and electron carriers (Ruiz-Sola et al. 2016a, b). For chlorophyll, tocopherol and phylloquinone synthesis, GGPP must be reduced to PhyPP by GGR. Accordingly, the reduction of GGR activity results in the accumulation of geranylgeranylated chlorophyll (chl_GG_) rather than the predominant phytylated form (chl_Phy_), lower overall chlorophyll and tocopherol levels, and greater sensitivity to light stress (Kimura et al. 2018; Liu et al. 2015; Tanaka et al. 1999; Wang et al. 2014; Zhou et al. 2013). The loss of GGR may also be associated with slower growth, a pale variegated phenotype, and restricted chloroplast development, which could reflect the differential expression of plastid-encoded genes (He et al. 2022; Liu et al. 2021; Tanaka et al. 1999). Consistent with their role in the formation of photosynthesis-related isoprenoids, GGRs are located in chloroplasts (Suire et al. 2000; Tanaka et al. 2010; Zhou et al. 2017) and transcript levels are highest in photosynthetically active tissues, but *GGR* genes are also expressed in fruits, roots, flowers and rice (*Oryza sativa*) bran (Bruno et al. 2009; Giannino et al. 2004; He et al. 2022; Kimura et al. 2018; Liu et al. 2012; Park et al. 2010; Zhou et al. 2013). Transcriptional regulation by phytohormones as well as temperature, light, drought, salt and biotic stress suggests a dependence on photosynthetic activity, ensuring a sufficient supply of chlorophyll and tocopherol (Bruno et al. 2009; Giannino et al. 2004; Liu et al. 2015; Park et al. 2010; Riva-Roveda et al. 2016; Zhou et al. 2013). GGR enzymes have not been studied in *Taraxacum* thus far, but one *GGR* gene was shown to be significantly downregulated in pale green leaves of *T. koksaghyz* plants overexpressing *pseudo-etiolated-in-light (PEL)-like* compared with near-isogenic control lines (Wolters et al. 2023).

The unexpected finding that a GGR may interact with TkSRPP3 in *T. koksaghyz* latex prompted the further characterization of this protein to shed light on its role in latex and its connection to NR biosynthesis and stress-related TkSRPP3. Therefore, we confirmed the enzymatic activity of the protein (designated TkGGR1), compared its molecular characteristics to a second TkGGR (TkGGR2), and investigated its interactions with associated proteins identified in other species. Based on our results, we propose the presence of a TkGGR1-containing heteromeric protein assembly within Frey–Wyssling (F.W.)-like complexes in the latex of *T. koksaghyz*, with a potential role in stress tolerance.

## Materials and methods

### Plant material and cultivation

We cultivated *T. koksaghyz*, *T. brevicorniculatum* and *Nicotiana benthamiana* plants under controlled greenhouse conditions (18 °C, 16-h photoperiod, 260 PPFD high-pressure sodium lamp with enhanced yellow and red spectrum) as previously described (Unland et al. 2018). To induce vernalization-dependent flowering, *T.* *koksaghyz* plants were transferred to a growth chamber at 6 °C for 2–3 weeks. For quantitative PCR (qPCR) analysis, leaf and root tissues were harvested separately and immediately flash-frozen in liquid nitrogen. Leaf tissues were ground using a pestle and mortar, with constant cooling. Root tissues were lyophilized and then pulverized using a ZM 200 Ultra Centrifugal Mill (Retsch, Germany). Latex was transferred from cut root surfaces to rubber extraction buffer (100 mM Tris–HCl pH 7.8, 350 mM sorbitol, 10 mM NaCl, 5 mM MgCl_2_, 5 mM dithiothreitol (DTT)) and immediately flash-frozen in liquid nitrogen.

### Generation of transgenic *T. koksaghyz* and *T. brevicorniculatum* plants and transgene verification

Transgenic *T.* *koksaghyz* and *T.* *brevicorniculatum* plants were generated as previously described with slight modifications (Niephaus et al. 2019; Post et al. 2012). For transformation, whole leaves were abaxially scratched and incubated with *Agrobacterium tumefaciens* strain EHA105, then placed on callus induction medium lacking antibiotics under constant light at 26 °C for 48 h, before transfer to medium supplemented with antibiotics. During regeneration, plants were incubated at 22 °C with a 16-h photoperiod. Transgene integration was confirmed by PCR using construct-specific primers.

### Cloning

Target genes were amplified from a mixture of wild-type *T.* *koksaghyz* cDNA (obtained from different tissues) using primers containing NcoI and NotI (*TkGGR1*, *TkGGPS6* and *TkLIL3*) or NcoI and XhoI (*TkGGR2*, *TkSRPP3* and *TkSRPP4*) restriction site overhangs (Supplementary Table 1) and transferred to the Gateway pENTR4 entry vector (Thermo Fisher Scientific, USA). The *TkGGR1* and *TkGGPS6* clones were also amplified without their predicted transit peptide (TP) sequences. TkSRPP constructs, and monomeric fluorophore mEmerald used as a negative control in protein interaction studies, were available from previous studies (Jekat et al. 2013; Wolters et al. 2024). For protein interaction studies and to assess the intracellular localization of the proteins, target genes were introduced into different Gateway destination vectors using Gateway LR clonase II mix (Thermo Fisher Scientific). For the split-ubiquitin membrane yeast two-hybrid (SUY2H) assays, target genes were introduced into pRS314-*Nua*-*ccdB* or pRS313-*ccdb*-*CRU* (Wolters et al. 2024). For bimolecular fluorescence complementation (BiFC) analysis, target genes were introduced into Gateway destination vectors containing the N-terminal or C-terminal part of monomeric red fluorescent protein (NmRFP or CmRFP): pBatTL-*ccdB*-*CmRFP*, pBatTL-*ccdB*-*NmRFP*, pBatTL-*NmRFP*-*ccdB*, or pBatTL-*CmRFP*-*ccdB* (Jach et al. 2006). To investigate intracellular localization in *N.* *benthamiana*, genes were introduced into the Gateway destination vector pBatTL-*ccdB*-*Cerulean* (Müller et al. 2010). To assess intracellular localization in *Saccharomyces cerevisiae*, the *TkGGR1* gene was introduced into pAGD425GDP-*ccdB*-*eCFP* from Addgene (Alberti et al. 2007). For transient expression in *N. benthamiana* and subsequent metabolic analysis, *TkGGR1∆TP* and *TkGGPS6(∆TP)* were introduced into pBatTL-*ccdb* destination vectors (Jach et al. 2006).

For *TkGGR1* overexpression in latex, the *T.* *brevicorniculatum rubber elongation factor* (*TbREF*) promoter (Laibach et al. 2015a, b) was inserted into pLab12.1 (Post et al. 2012) using the restriction sites HindIII and XhoI. The cauliflower mosaic virus 35S terminator was isolated from vector pAM (Fricke et al. 2013) using SacI and BamHI and inserted into the newly generated pLab12.1-P_REF_ (digested with the same enzymes). Finally, *TkGGR1* was amplified using primers with XhoI and XbaI restriction sites and inserted into the intermediate vector (digested with the same enzymes) resulting in the final plant transformation vector pLab12.1-P_REF_-*TkGGR1*-T_35S_. The *TkGGR1*–RNAi construct was prepared by annealing the oligonucleotides *TkGGR1*-RNAi-NcoI and *TkGGR1*-RNAi-XhoI, and the double-stranded product with NcoI and XhoI sites was ligated into the corresponding sites of the Gateway entry vector pBluescript II KS (+) (Addgene). The resulting pBluescript-*TkGGR1*-RNAi vector was used for Gateway cloning into the destination vector pLab12.5 (Epping et al. 2015). The resulting pLab12.5-p_REF_-*TkGGR1*-RNAi-T_OCS_ vector was used for dandelion transformation.

For the *TbGGR2*–RNAi construct, the oligonucleotides *GGR2* RNAi NcoI fw and *GGR2* RNAi XhoI rv were annealed, and the double stranded product with NcoI an XhoI sites was ligated into corresponding sites in the vector pENTR4 to yield pENTR4-*TbGGR2*-RNAi. This was used as the entry clone in a Gateway reaction with pFGC5941 (ChromDB, USA) yielding the final plant transformation vector pFGC-*TbGGR2*-RNAi. For mVenus-tagged TkGGR1 expression in latex, *TkGGR1* was amplified without the stop codon using primers containing NcoI and XbaI sites. The pLab12.1-P_REF_-T_35S_ vector was digested with the same restriction enzymes and *TkGGR1* was inserted. In a second step, mVenus was amplified from pFRETtv-2in1 (Hecker et al. 2015) using primers containing XbaI and XmaI sites and inserted at the corresponding sites of pLab12.1-P_REF_-*TkGGR1*-T_35S_.

### In silico analysis

Phylogenetic analysis was carried out using MEGA11 (Tamura et al. 2021). The multiple sequence alignment was created using MUSCLE and the phylogenetic tree was constructed using the neighbor-joining algorithm with a bootstrap of 462. Protein domains were predicted using InterPro (Paysan-Lafosse et al. 2023). Sequences were aligned using Clustal Omega (Madeira et al. 2024). TPs were predicted using TargetP-2.0 (https://services.healthtech.dtu.dk/services/TargetP-2.0/).

### RNA extraction, cDNA synthesis and qPCR

RNA extraction, cDNA synthesis and qPCR were carried out as previously described (Wolters et al. 2024).

### Chlorophyll quantification

Chlorophyll was extracted as previously described with slight modifications (Shpilyov et al. 2005). Briefly, a leaf disc was placed in a microfuge tube and homogenized with 1 mL 90% methanol in water using an MM400 bead mill (Retsch) for 5 min at 30 Hz, followed by centrifugation at 14,000 g for 1 min. The supernatant was transferred to a new tube and the extraction step was repeated. The extracts were separated by high performance liquid chromatography (HPLC) using a Shimadzu LC20A UFLC prominence system (Shimadzu, Germany) fitted with a Reprosil Pur Basic C18 column (Analytik Altmann, Germany; 5 µm particle size, 4 × 250 mm) coupled to an SPD-M20A photodiode array (PDA) detector. Samples were eluted in a gradient of solvent A (900:99:1 *v*/*v* acetonitrile: water: triethylamine) and solvent B (ethyl acetate): 100% A for 16 min, 66.7% A for 8 min, 59.7% A for 16 min, 33.3% A for 0.2 min, 0% A for 2.5 min, 100% A for 3.25 min. The flow rate was 1 mL/min, the column oven temperature was 40 °C. Pigments were identified based on their absorption spectra (430 nm for chlorophyll *a* and 458 nm for chlorophyll *b*) and relative retention times.

### Heterologous expression in *N. benthamiana*

For transient expression in *N. benthamiana* leaf epidermal cells, we used pBatTL constructs carrying either the target gene fused to the full-length fluorophores Cerulean or Venus, or the target gene fused to the N-terminal or C-terminal part of monomeric red fluorescent protein, NmRFP or CmRFP (Jach et al. 2006; Müller et al. 2010). Transient expression was achieved as previously described (Müller et al. 2010). Different constructs were co-expressed with the Cerulean-fused genes. We used Arabidopsis (*Arabidopsis thaliana*) *LEAFY COTYLEDON 2* (*AtLEC2*) for the induction of lipid droplet (LD) formation (Wolters et al. 2024), and the chloroplast TP of tobacco (*Nicotiana tabacum*) d-ribulose-1,5-bisphosphate carboxylase/oxygenase (RuBisCO) C-terminally fused to Venus as a stromal marker. The sequence of the tobacco RuBisCO TP was amplified from *N. tabacum* SR1 cDNA (Schmidt et al. 2020). Primers introducing NcoI and NotI restriction sites were used and the product was inserted into pENTR4. The resulting vector was used for Gateway cloning into the pBatTL-*ccdB*-*Venus* destination vector. For bimolecular fluorescence complementation (BiFC), all eight combinations of NmRFP/CmRFP fusions of two target proteins were co-expressed followed by fluorescence microscopy.

### Heterologous expression in *S. cerevisiae*

Protein interaction studies based on SUY2H were carried out as previously described (Wolters et al. 2024). For localization studies, *S. cerevisiae* strain CEN.PK2-1C (Westfall et al. 2012) (EUROSCARF, Germany) was transformed using the lithium acetate method (Agatep et al. 1998). Positive clones were identified by colony PCR using gene-specific and vector primers. They were grown at 30 °C on selective synthetic defined media, transferred to a microscope slide and suspended in water. To stain LDs, the suspension was supplemented with 1 µg Nile red in DMSO.

### Microscopy

Confocal laser scanning microscopy was carried out using a Stellaris 8 or TCS SP5 X microscope (Leica Microsystems, Germany). Cerulean fluorescence was detected at 470–518 nm (excitation at 405, 448 or 458 nm), mRFP fluorescence at 570–648 nm (excitation at 515 or 555 nm), Venus fluorescence at 556–615 nm (excitation at 488 or 514 nm), mVenus fluorescence at 520–650 nm (excitation at 515 nm), eCFP fluorescence at 469–502 nm (excitation at 405 nm), Nile red fluorescence at 556–615 nm (*N.* *benthamiana*) or 570–603 nm (*S. cerevisiae*) (excitation at 488 nm), and mEmerald fluorescence at 501–550 nm (excitation at 481 nm). Chlorophyll autofluorescence was detected at 655–752 nm (excitation at 448–458 nm).

### Immunodetection of fluorophore fusion proteins from latex

Latex phase separation was carried out as previously described (Niephaus et al. 2019). Latex phases were mixed with 5 × SDS loading buffer containing 100 mM DTT, heated at 95 °C for 10 min and separated by SDS-PAGE (Wolters et al. 2024). For immunodetection, we used a primary anti-GFP antibody (Clontech Laboratories, USA; #632,380) and a secondary goat antimouse IgG antibody coupled to horseradish peroxidase (Thermo Fisher Scientific; #32430).

### Quantification of phytol and geranylgeraniol in *N. benthamiana*

Infiltrated leaves were harvested, immediately flash-frozen and pulverized under liquid nitrogen with a pestle and mortar. We then extracted 100 mg freeze-dried, pulverized leaf material supplemented with 250 µg betulin (internal standard) with 1 mL ethyl acetate by vortexing for 10 min. After centrifugation at 11,000 g for 5 min, the extracts were evaporated and the residue redissolved in 500 µL hexane. The samples were analyzed by gas chromatography–mass spectrometry (GC–MS) using a GC–MS-QP 2010 Ultra High-End device (Shimadzu Corporation, Japan) fitted with an Rtx-5 ms column (Restek, Germany; 30 m × 0.23 mm, film thickness 0.25 µm) and helium as the mobile phase. Electron ionization and a quadrupole mass filter system were used. The injection volume was 0.5–1 µL at a temperature of 260 °C in split mode (1:10). The ion source temperature was 230 °C and the interface temperature 260 °C. The GC program included 1 min at 120 °C followed by a steep ramp to 330 °C at 15 °C/min and a final step at 330 °C for 10 min. Mass spectra were evaluated based on authentic standards and the National Institute of Standards and Technology (NIST) libraries using LabSolution software (Shimadzu). Quantification was performed relative to the internal standard betulin.

### Quantification of triterpenoids, precursors, tocopherols, GGOH and NR in dandelion

For triterpenoids, precursors and tocopherol, 100 mg freeze-dried, pulverized root material was supplemented with 250 µg betulin (internal standard) and extracted and saponified with 20 mL methanol containing 6% potassium hydroxide at 80 °C for 2 h. Evaporated methanol was replaced with water up to a volume of 15 mL, and metabolites were extracted with 15 mL hexane by vigorous vortexing and subsequent phase separation by centrifugation at 4000 g for 5 min. The hexane phase was transferred to another tube and the extraction step was repeated twice. The hexane phase was evaporated in a Rocket Evaporator System (Thermo Fisher Scientific) and dissolved in 1.2 mL acetone overnight at 40 °C. After brief centrifugation (11,000 g, 2 min), 500 µL of the supernatant was removed for GC–MS analysis. The GC–MS protocol was the same as described for phytol and geranylgeraniol analysis. Mass spectra were evaluated based on NIST libraries or authentic standards, and analytes were quantified relative to the internal standard. NR was quantified as previously described (Stolze et al. 2017).

## Accession numbers

*TkGGPS6*, GWHTAAAA038209; *TkLIL3*, GWHTAAAA016040; *TkSRPP3*, GWHTAAAA015362; *TkSRPP4*, GWHTAAAA015361 (Lin et al. 2018). For *TkGGR* accession numbers, refer to Supplementary Table 2.

## Results

### Confirmation of TkGGR1/TkSRPP3 protein interaction

**Fig. 1:**
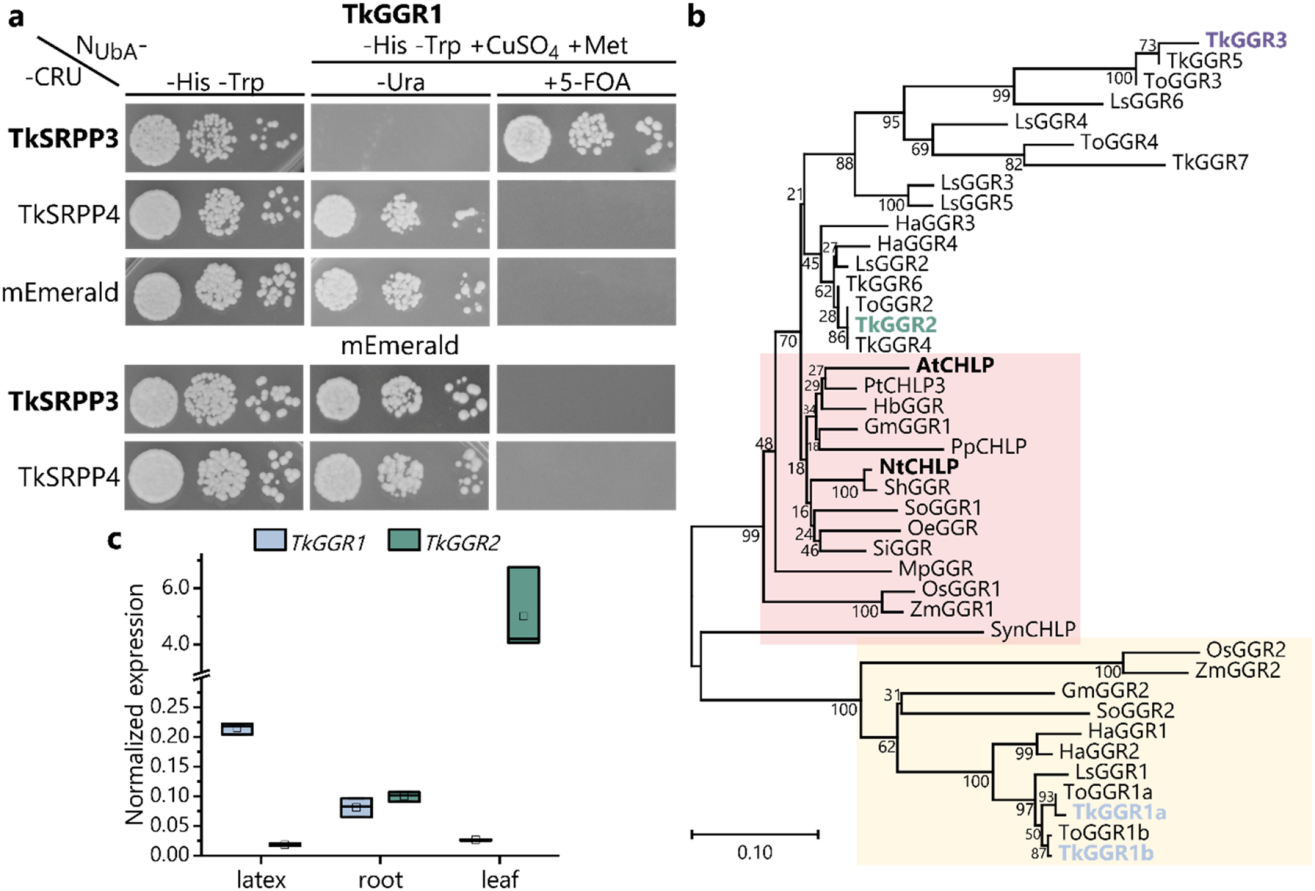
Characterization of TkGGR1, which interacts with TkSRPP3. **a** Split-ubiquitin membrane yeast-two hybrid (SUY2H) assay confirming a specific interaction between TkGGR1 and TkSRPP3. Yeast expressing TkGGR1 N-terminally fused to the N-terminal part of ubiquitin (N_UbA_) and TkSRPP3 C-terminally fused to the ubiquitin C-terminus and the URA3 reporter (CRU) were dropped in three different dilutions onto selective media and grown for 2–3 days. Medium without histidine and tryptophan (–H–T) was used as a control only selecting for the plasmids encoding both fusion proteins. Medium also lacking uracil and containing 50 µM CuSO_4_ and 300 µM methionine (–H–T–U + CuSO_4_ + M) was used to select for URA3 activity. Medium containing uracil and 1 g/L 5-FOA (–H–T + CuSO_4_ + M + 5-FOA) was used to select for URA3 inactivity, reflecting protein interactions. TkSRPP4 and the fluorophore mEmerald were used as negative controls. **b** Phylogenetic analysis showing separate clustering of TkGGR1a/b with OsGGR2 and uncharacterized proteins (yellow box) separated from well-described homologs (red box). TkGGRs 2, 4 and 6 were most closely related to the characterized GGRs (red box). Bootstrap values are indicated next to the branches. The phylogenetic distance is indicated by the scale bar. Accession numbers: AtCHLP, *Arabidopsis thaliana* CHLP (Q9CA67); GmGGR1/2, *Glycine max* GGR1/2 (A0A445G4S7; K7KB36); HaGGR1/2/3/4, *Helianthus annuus* GGR1, 2, 3 & 4 (A0A251V8X1; A0A251TZT8; KAJ0766007.1; A0A251US85); HbGGR, *Hevea brasiliensis* GGR (B7X936); LsGGR1/2/3/4/5/6, *Lactuca sativa* GGR1, 2, 3, 4, 5 & 6 (XP_023747237.1; XP_023761285.1; XP_023730011.1; XP_023730009.1; XP_023730008.1; XP_023730032.1); MpGGR, *Marchantia polymorpha* GGR (BFI34017.1); NtCHLP, *Nicotiana tabacum* CHLP (Q9ZS34); OeGGR, *Olea europaea* GGR (A0A8S0TCV4); OsGGR1/2, *Oryza sativa* GGR1/2 (Q6Z2T6; A0A0P0V137); PpCHLP, *Prunus persica* CHLP (AAP55675.1); PtCHLP3, *Populus trichocarpa* GGR3 (B9I2Y8); ShGGR, *Solanum habrochaites* GGR (AIU39220.1); SiGGR, *Sesamum indicum* GGR (E2D5V0); SoGGR1/2, *Spinacia oleracea* GGR1/2 (XP_021860416.1; XP_021859495.1); SynCHLP, *Synechocystis* sp. (Q55087); TkGGR1/2/3/4/5/6/7, *T.* *koksaghyz* GGR1, 2, 3, 4, 5, 6 & 7 (for accession numbers, see Supplementary Table 2); ToGGR1/2/3/4, *T. officinale* GGR1, 2, 3 & 4 (sequences from internal data); ZmGGR1/2, *Zea mays* GGR1/2 (B6TDR5; A0A8J8XXZ5). **c** *TkGGR1* expression is latex-specific and *TkGGR2* expression is leaf-specific. Normalized gene expression in different tissues of 12-week-old wild-type *T.* *koksaghyz* plants obtained by qPCR. No *TkGGR3* transcripts could be detected by qPCR. Box plots represent data from three pools consisting of cDNA from four individual plants. *TkGGR1* expression is the sum of transcripts from both gene copies. Expression levels were normalized against *elongation factor-1 α* (*TkEF1α*) and *ribosomal protein L27* (*TkRP*)

First, we attempted to confirm the interaction between TkSRPP3 and TkGGR1 initially indicated by affinity enrichment-mass spectrometry (AE–MS) using the split-ubiquitin membrane yeast two-hybrid system (SUY2H), which is ideal for detecting interactions between non-nuclear hydrophobic or membrane-associated proteins. The C-terminus of TkSRPP3 was fused to the C-terminal part of ubiquitin (C_Ub_) and the URA3 reporter, and was co-expressed with TkGGR1 N-terminally fused to a modified ubiquitin N-terminus (N_UbA_) with lower affinity for C_Ub_ (Johnsson and Varshavsky 1994). Protein interaction leads to the degradation of URA3 in the cytosol, resulting in uracil auxotrophy and 5-fluoroorotic acid (5-FOA) tolerance (Johnsson and Varshavsky 1994; Reichel and Johnsson 2005). This confirmed a specific reaction between TkGGR1 and TkSRPP3, but not TkSRPP4 and mEmerald, which were used as negative controls (Fig. 1a).

### Identification of the *GGR* gene family in *T. koksaghyz*

Given the presence of multiple *GGR* genes in other plants such as rice and poplar (He et al. 2022; Kimura et al. 2018), we searched the *T. koksaghyz* genome for additional *GGR* genes, which could be compared with *TkGGR1*. Using our AE–MS data, we identified the *TkGGR1* sequence in the genome assembly published in 2018 (Lin et al. 2018; Wolters et al. 2024). The sequence was used as a BLAST query against the most recently published genome assembly comprising pseudo-chromosomes (Lin et al. 2022), revealing the presence of two *GGR1* gene copies with high sequence identity (95.85%) on pseudo-chromosomes 4 and 6. However, the *TkGGR1* copy on pseudo-chromosome 6 contained a premature stop codon after 228 bp. The genome assembly published in 2018 also contained two *GGR* sequences with 96.67% nucleotide sequence identity, both encoding full-length proteins (97% protein sequence identity). One most likely corresponds to the gene copy on pseudo-chromosome 4 (99.71% identity) and the other to the copy on pseudo-chromosome 6 (98.56%) that may have undergone mutation in the sequenced plant, probably resulting in a loss of function. The two variants identified in the 2018 genome assembly were designated *TkGGR1a* and *TkGGR1b*, and the *TkGGR1a* sequence was used in all subsequent experiments. A recent transcriptomic comparison of *T.* *koksaghyz* plants overexpressing *TkPEL-like* with wild-type controls identified another *GGR* sequence, designated *TkGGR3*, which was significantly downregulated in the leaves of the transgenic plants (Wolters et al. 2023). BLAST searches against both available *T.* *koksaghyz* genomes with *CHLP*, a well-characterized tobacco *GGR* (Tanaka et al. 1999), led to the identification of five additional putative *GGR* genes, some of which were partial sequences of others (Supplementary Table 2). Phylogenetic analysis of the seven putative TkGGR proteins and sequences from other species revealed distinct clustering of the paralogs (Fig. 1b). TkGGR1a/b clustered together with other putative enzymes and rice GGR2, which is expressed in leaves and bran and is required for tocopherol synthesis (Kimura et al. 2018), but other well-characterized GGRs formed a separate cluster. Most of the well-characterized GGRs, including those from tobacco and Arabidopsis, clustered together along with TkGGRs 2, 4 and 6, whereas TkGGRs 3, 5 and 7 clustered separately (Fig. 1b).

### *TkGGR1 *is predominantly expressed in latex whereas *TkGGR2* expression is leaf-specific

Internal RNA-Seq data indicated high-level *TkGGR2* expression in leaves, which suggested this paralog is the main enzyme responsible for GGPP reduction during chlorophyll and tocopherol biosynthesis in chloroplasts. Therefore, we selected this paralog together with the earlier identified *TkGGR3* for comparative analysis with *TkGGR1*. Initially, we performed qPCR using cDNA obtained from different tissues of 12-week-old wild-type *T. koksaghyz* plants. No discriminating primers could be designed for the two *TkGGR1* gene copies so the recorded mRNA levels reflect the sum of both genes. *TkGGR1* was expressed most strongly in the latex, with very low levels in leaves, whereas *TkGGR2* was predominantly and strongly expressed in leaves, in agreement with the internal RNA-Seq data (Fig. 1c). For *TkGGR3*, we could not detect transcript levels above background in any of the tested tissues. According to these profiles, TkGGR2 may facilitate photosynthesis-related isoprenoid synthesis and TkGGR1 probably has a different function in latex. In silico protein domain prediction indicated that TkGGR1 and TkGGR2, but not TkGGR3, contain a NAD- or NAD/FAD-binding domain important for the catalysis of redox reactions, as also found in AtCHLP and NtCHLP (Supplementary Fig. 10).

### *TkGGR2* silencing confers a pale green phenotype, chl_GG_ accumulation and reduced biomass

**Fig. 2:**
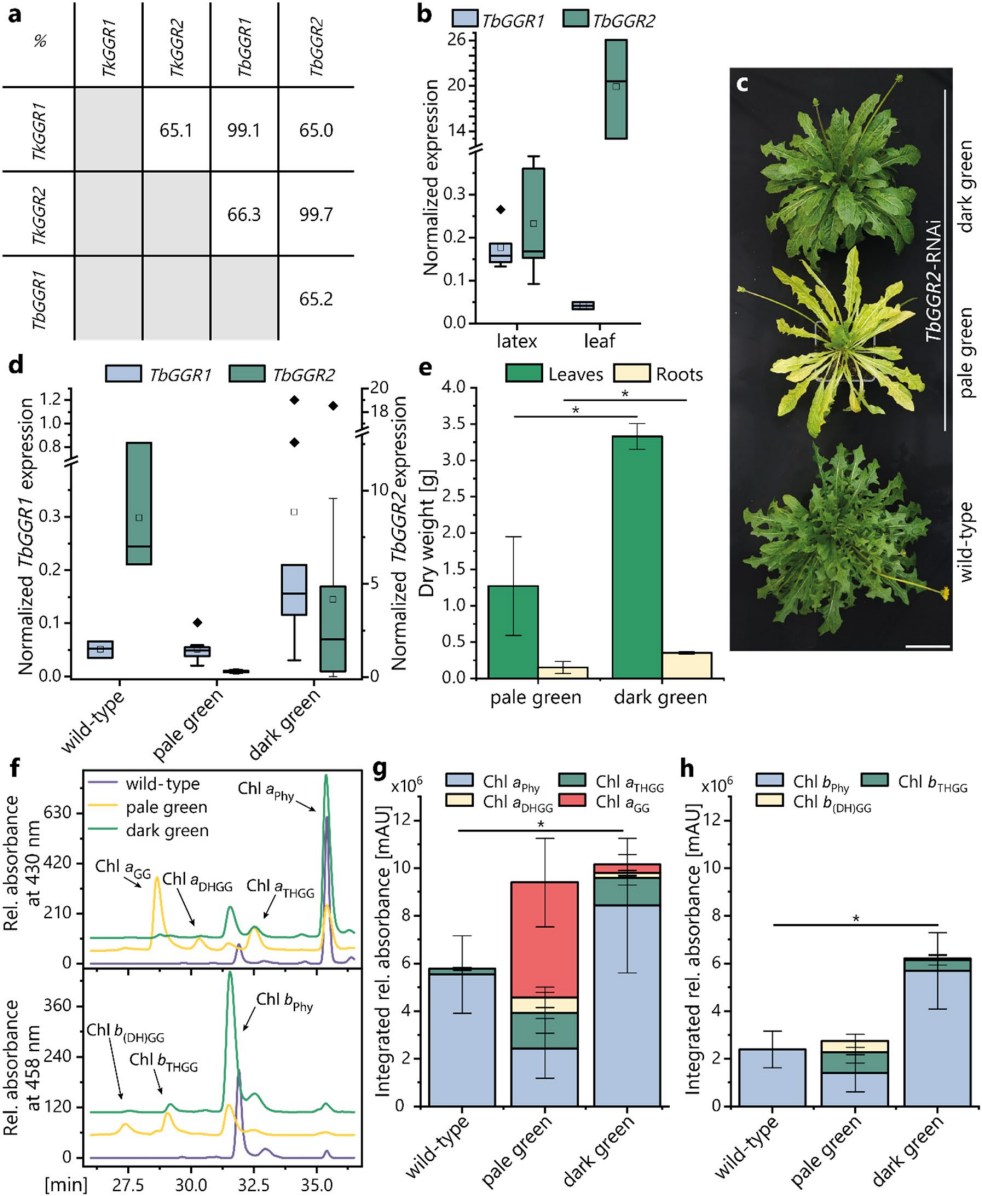
Strong *TbGGR2* knockdown results in a pale green leaf phenotype, reduced leaf and root biomass, and the accumulation of chl_GG_. **a** DNA sequence comparison of *Tk/TbGGR1/2*. Strong conservation between the species highlights the suitability of *T.* *brevicorniculatum* as a model. **b** *TbGGR1/2* expression in latex and leaves is similar to the orthologs in *T.* *koksaghyz*. Data are normalized expression levels in 14-week-old wild-type *T. brevicorniculatum* plants. Box plots represent data from three (leaves) or five (latex) individual plants. Expression levels were normalized against *elongation factor-1 α* (*TkEF1α*) and *ribosomal protein L27* (*TbRP*). **c** Phenotypes of two types of *TbGGR2*-RNAi lines with either pale green or normal dark green leaves in comparison to a wild-type *T.* *brevicorniculatum* plant. The plants were grown under controlled greenhouse conditions for 14 weeks. Scale bar = 10 cm. **d** *TbGGR2* expression is strongly reduced in the leaves of pale green and moderately reduced in the leaves of dark green *TbGGR2*-RNAi lines. *TbGGR1* expression is slightly increased in the leaves of dark green *TbGGR2*-RNAi lines compared to wild-type controls. Box plots represent normalized gene expression in leaves of three wild-type, eight pale green and 10 dark green *TbGGR2*-RNAi plants. Expression levels were normalized against *TbEF1α* and *TbRP*. **e** Pale green *TbGGR2*-RNAi plants have significantly reduced leaf and root biomass compared to dark green *TbGGR2*-RNAi plants. Plants were grown under controlled greenhouse conditions for 8 weeks before leaves and roots were harvested, dried and quantified. Data represent means of five individuals of three independent lines for both phenotypes (*n* = 15) ± standard deviations. Statistical significance was calculated using the Wilcoxon signed ranks test (*p < 0.05). **f** HPLC chromatograms of chlorophyll extracts from pale green *TbGGR2*-RNAi plants show three additional peaks for chlorophyll *a* (chl *a*_THGG_, chl *a*_DHGG_ and chl *a*_GG_) and two additional peaks for chlorophyll *b* (chl *b*_THGG_ and chl *b*_(DH)GG_) compared to wild-type controls. **g**, **h** Quantification of chlorophylls *a* (**g**) and *b* (**h**) and their composition in wild type, pale green and dark green *TbGGR2*-RNAi plants. Dark green *TbGGR2*-RNAi plants have higher total chlorophyll *a* and* b* levels. Pale green *TbGGR2*-RNAi plants contain a high proportion of chlorophylls lacking fully reduced prenyl side chains that are almost completely absent in the wild-type leaves. Values are means of three wild type, eight pale green and 10 dark green *TbGGR2*-RNAi plants ± standard deviations. Statistical significance was calculated using two-sample* t* tests (*p < 0.05) (Colour figure online)

**Fig. 3:**
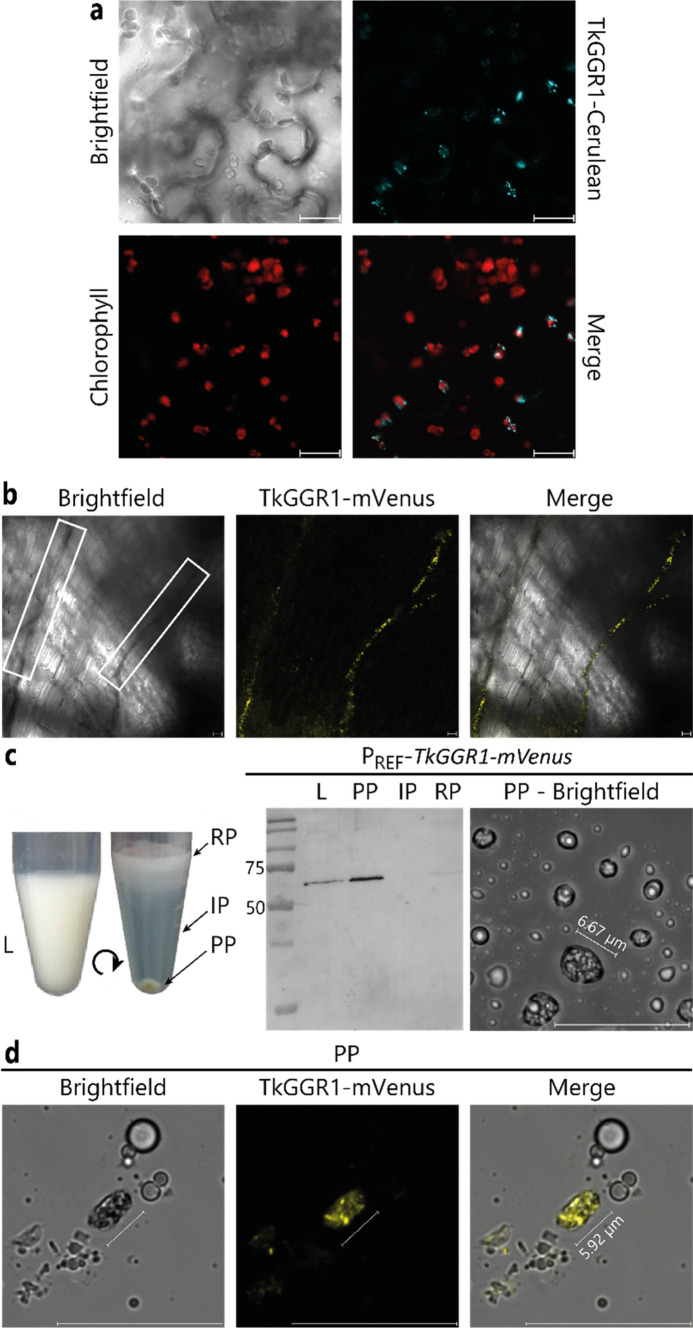
TkGGR1 is found in punctuate structures within leaf chloroplasts and is imported into plastid-like structures of the latex pellet phase. **a** Punctuate TkGGR1–Cerulean fluorescence (cyan) within chloroplasts marked by chlorophyll autofluorescence in *N.* *benthamiana*. Microscopic images of *N.* *benthamiana* leaf epidermal cells transiently expressing a TkGGR1–Cerulean fusion protein, with chlorophyll autofluorescence shown in red. **b** TkGGR1–mVenus fluorescence in the laticifers of P_REF_-*TkGGR1-mVenus* roots. A longitudinal root section is shown and laticifers are enclosed by white boxes. **c** Latex (L) can be separated into three phases by centrifugation: the floating rubber phase (RP), the cytosolic interphase (IP) and the bottom pellet phase (PP). TkGGR1-mVenus segregates into the PP after latex centrifugation. Immunodetection of the TkGGR1-fused mVenus indicated the presence of the fusion protein in whole latex and its concentration in the PP. A weak signal is also visible in the RP. Molar mass in kDa of the protein standard is indicated by numbers. Microscopic images show the presence of 6–7 µm long oval particles within the PP. **d** TkGGR1–mVenus fluorescence is located inside plastid-like particles in the PP. Scales bars = 20 µm

The distinct spatial expression profiles of *TkGGR1* and *TkGGR2* and their low nucleotide sequence identity of only 65% (Fig. 2a) suggested functional divergence. To determine whether TkGGR2 is responsible for the reduction of GGPP for chl_Phy_ synthesis in chloroplasts, we used RNA interference (RNAi) to generate transgenic *T.* *brevicorniculatum* plants with reduced *GGR2* expression. This dandelion species was used because it produces more homogenous progeny due to its apomictic propagation (Kirschner et al. 2013) and has a higher transformation efficiency than *T. koksaghyz*. The *T.* *brevicorniculatum* and *T. koksaghyz GGR2* genes show > 99% nucleotide sequence identity, highlighting the suitability of *T. brevicorniculatum* for this experiment (Fig. 2a). Initial analysis of *TbGGR1* and *TbGGR2* expression confirmed similar expression patterns to their *T.* *koksaghyz* orthologs, although tissue-specific expression was not as distinctive because relatively high levels of *TbGGR2* mRNA were detected in latex (Fig. 2b). Two types of positive transformants were recovered, one with a wild-type (dark green) leaf phenotype and one with a pale green leaf phenotype (Fig. 2c, Supplementary Fig. 10). Using qPCR, we showed that *TbGGR2* mRNA levels were strongly reduced in the pale green leaves and moderately reduced in the dark green leaves of transgenic plants as compared to *T. brevicorniculatum* wild-type controls (Fig. 2d). *TbGGR1* expression levels were comparable in pale green and wild-type leaves, but tended to be higher in the dark green leaves of *TbGGR2*-RNAi plants (Fig. 2d, Supplementary Fig. 11). This indicates a relationship between the leaf phenotype and the *TbGGR1/TbGGR2* expression level. The pale green *TbGGR2*-RNAi plants also appeared to be smaller than their dark green counterparts, so we quantified the root and leaf biomass of these plants. This revealed a significant reduction in the dry weight of both tissues in 8-week-old pale green as compared to dark green *TbGGR2*-RNAi lines (Fig. 2e). We also measured chlorophyll levels in the leaves of both phenotypes as compared to wild-type controls. HPLC chromatograms of leaf extracts from pale green *TbGGR2*-RNAi plants showed three additional peaks for chlorophyll *a* compared to wild-type plants, representing chl *a*_GG_ and the intermediates chl *a*_THGG_ and chl *a*_DHGG_ (Fig. 2f). For chlorophyll *b*, two additional peaks were identified for the pale green *TbGGR2*-RNAi leaves. The one at 29 min probably represents chl *b*_THGG_ whereas the one at 26.9 min probably represents both chl *b*_DHGG_ and chl *b*_GG_ (Fig. 3f). We observed significantly increased chl *a* and *b* levels in dark green *TbGGR2*-RNAi plants compared to wild-type controls (Fig. 2g, h). In contrast, the pale green leaves contained only 25% chlorophyll *a* and 50% chlorophyll *b* esterified with phytol whereas wild-type chlorophyll was made up almost exclusively of chl *a*_Phy_ and chl *b*_Phy_. The dark green leaves showed an intermediate pattern, with ~ 83% chl *a*_Phy_ and ~ 91% chl *b*_Phy_ (Fig. 2g, h). These findings further substantiated that the phenotype depends on the extent of transcriptional downregulation and provided strong evidence that TbGGR2 is responsible for GGPP reduction during chlorophyll synthesis.

### *TkGGR1 *has affinity for different lipids and is located within uncharacterized particles in the latex pellet phase of *T. koksaghyz*

Unlike *TkGGR2*, *TkGGR1* is barely expressed in leaves. However, GGRs are reported to be localized within chloroplasts, so the question raised is whether TkGGR1 is also translocated to these organelles. To address this question, we fused the C-terminus of TkGGR1 to the fluorescent reporter Cerulean for transient expression in *N. benthamiana* leaves. Cerulean fluorescence partially overlapped with the autofluorescence of chlorophyll, which marks the abundant chloroplasts (Fig. 3a). The TkGGR1-Cerulean signal within the chloroplasts was punctate, suggesting localization in the thylakoid membranes or plastoglobuli, which are lipoprotein particles inside chloroplasts (Lundquist et al. 2012; Vidi et al. 2006). This pattern was similar to that observed for the TkGGR2–Cerulean fusion (Supplementary Fig. 12). We considered the possibility that TkGGR1 shows a general affinity for lipid droplets (LDs), which share structural similarities with rubber particles. Accordingly, we expressed a TkGGR1–eCFP fusion protein in yeast, which produces abundant LDs. We found that the eCFP signal overlapped with that of the lipophilic dye Nile red, which stains LDs, indicating that TkGGR1 has an affinity for LDs in yeast (Supplementary Fig. 11a, b). Interestingly however, TkGGR1 lacking the chloroplast TP was not imported into chloroplasts but did not associate with cytosolic LDs in *N.* *benthamiana* (Supplementary Fig. 13c).

Given that *TkGGR1* is expressed in *T. koksaghyz* latex but not leaves, we further sought to investigate its localization in latex-bearing laticifers. We generated transgenic plants expressing a *TkGGR1–mVenus* fusion under the control of the *T.* *brevicorniculatum REF* promoter (P_REF_), which confers predominant expression in latex (Benninghaus et al. 2020; Laibach et al. 2015a, b). Analysis of the roots by fluorescence microscopy revealed patchy mVenus signals within the laticifers (Fig. 3b). To determine the localization of the TkGGR1–mVenus fusion protein in more detail, latex was harvested and centrifuged to separate the floating rubber phase from the aqueous interphase and bottom pellet phase (Fig. 3c). Proteomic analysis confirmed that the rubber phase mainly contains the NR-encapsulating rubber particles, the interphase mainly represents the cytosolic fraction, and the pellet phase contains organelles and membrane fragments (Wolters et al. 2024). Immunodetection of the TkGGR1-fused mVenus revealed the presence of the fusion protein in whole latex and the pellet phase, with a weak band also in the rubber phase (Fig. 3c). The proteins had an apparent mass of ~70 kDa, most likely corresponding to the TkGGR1–mVenus fusion without the TP, given that the mass of the full-length fusion protein is predicted to be 78 kDa and that of the truncated fusion protein 72 kDa. Microscopic analysis of the pellet phase revealed oval, irregularly filled particles 3.5–7 µm in length (Fig. 3c) and mVenus fluorescence of these particles indicated the presence of the TkGGR1–mVenus fusion protein (Fig. 3d).

### Latex-specific *TkGGR*1 overexpression and knockdown affects tocopherol and specific triterpenoid levels in latex-bearing roots

**Fig. 4:**
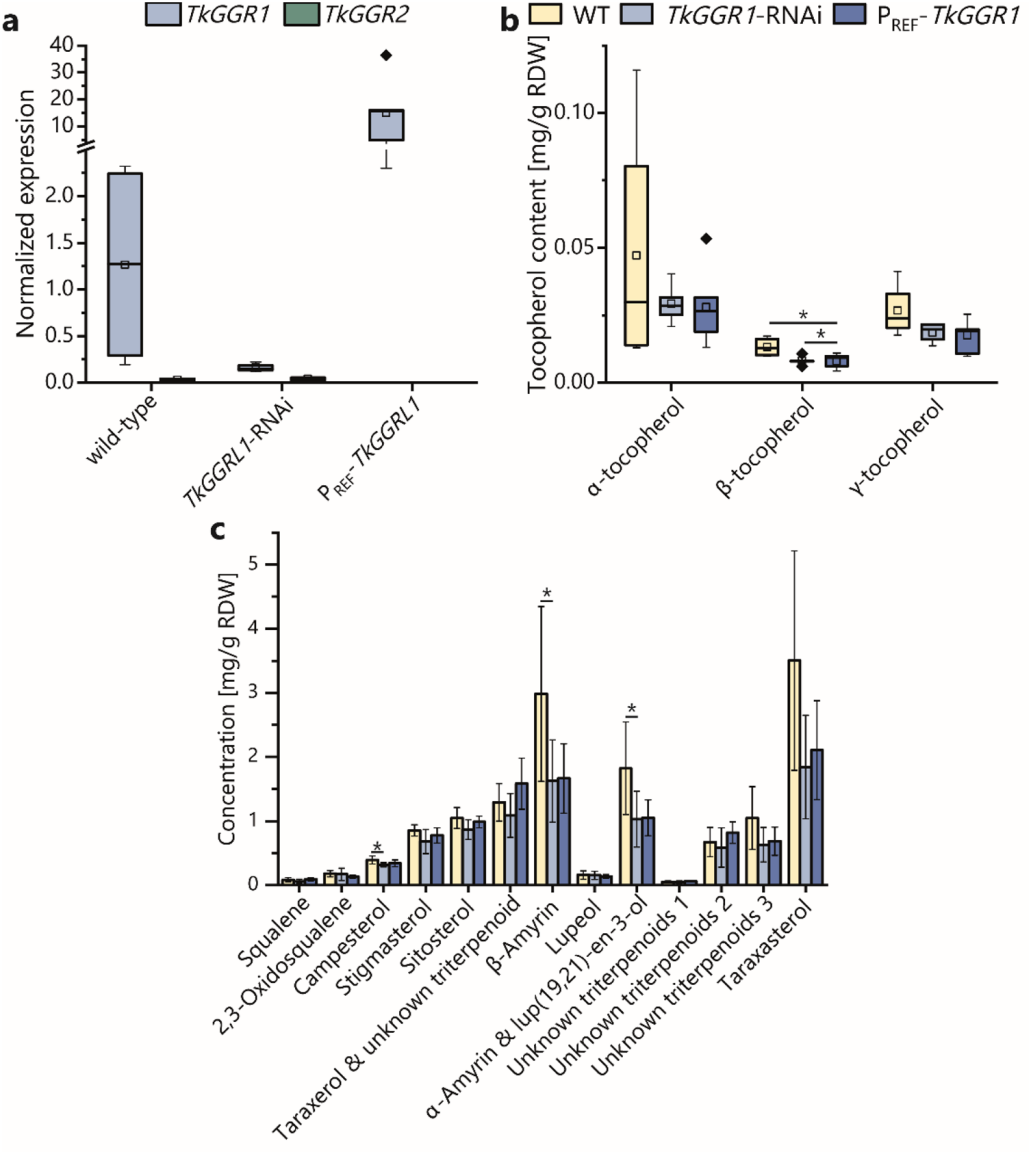
Depletion of β-tocopherol, lup(19,21)-en-3-ol, α-amyrin, β-amyrin and campesterol in the roots of *T.* *koksaghyz* lines with modified *TkGGR1* expression. **a** *TkGGR1* expression levels are reduced in latex of *T.* *koksaghyz TkGGR1*-RNAi plants, whereas *TkGGR2* expression remains unaffected. In P_REF_-*TkGGR1* lines, *TkGGR1* is strongly overexpressed compared to wild-type controls. Normalized gene expression levels in 14-week-old *T.* *koksaghyz* plants. Boxplots represent values from multiple individuals: wild-type *TkGGR1* *n* = 4, *TkGGR2* n = 2; *TkGGR1*-RNAi *n* = 7, *Tk* P_REF_-*TkGGR1* n = 5. In P_REF_-*TkGGR1* lines, only *TkGGR1* expression was examined. Expression levels were normalized against *elongation factor-1 α* (*EF1α*) and *ribosomal protein L27* (*RP*). **b** Root β-tocopherol levels are reduced in 14-week-old *TkGGR1*-RNAi and P_REF_-*TkGGR1* lines compared to wild-type *T.* *koksaghyz* controls. Statistical significance was determined using two-sample* t* tests (*p < 0.05). **c** Lup(19,21)-en-3-ol, α-amyrin, β-amyrin and campesterol are less abundant in roots of *T.* *koksaghyz TkGGR1*-RNAi plants compared to wild-type controls. Quantification of triterpenoids and phytosterols in roots of 14-week-old *T. koksaghyz* wild-type, *TkGGR1*-RNAi and P_REF_-*TkGGR1* plants. The data are means ± standard deviations. Statistical significance was determined using two-sample* t* tests, or the Wilcoxon signed ranks test when data were not normally distributed (*p < 0.05). Metabolites were quantified in multiple individual plants from different lines: wild-type *n* = 4, *TkGGR1*-RNAi *n* = 7, *Tk* P_REF_-*TkGGR1* *n* = 5

For functional analysis, we generated transgenic *T. koksaghyz* and *T. brevicorniculatum* plants expressing either *TkGGR1* or *GGR1*–RNAi constructs under the control of P_REF_, resulting in *GGR1* overexpression or knockdown in the latex. *Tk/TbGGR1* and *Tk/TbGGR2* mRNA levels in the latex of these plants were initially monitored by qPCR, and plants showing the strongest effect on *GGR1* but not *GGR2* expression were selected for metabolic profiling (Fig. 4a, Supplementary Fig. 9a). To assess the impact of GGR1 on secondary isoprenoids, we quantified the levels of tocopherols, NR, triterpenoids and their precursors in the roots of transgenic plants and wild-type controls (Fig. 4, Supplementary Figs. 14, 15). Although there was some metabolic heterogeneity among the plants, we observed a significantly lower β-tocopherol content and slight reductions in the levels of α- and γ-tocopherol in *T.* *koksaghyz TkGGR1*-RNAi and P_REF_-*TkGGR1* lines compared to wild-type controls (Fig. 4b). In *T.* *brevicorniculatum*, tocopherol levels were much lower and a corresponding effect was absent (Supplementary Fig. 14). The *T.* *koksaghyz TkGGR1*-RNAi plants also produced lower amounts of the phytosterol campesterol and the triterpenoids lup(19,21)-en-3-ol, α-amyrin and β-amyrin. Several other triterpenoids also tended to be depleted in the *T.* *koksaghyz TkGGR1*-RNAi and P_REF_-*TkGGR1* lines (Fig. 4c). In contrast, the *T.* *brevicorniculatum* P_REF_-*TkGGR1* lines were significantly enriched for two uncharacterized triterpenoids compared to wild-type plants (Supplementary Fig. 14c). NR levels were not affected in any of the tested plants (Supplementary Fig. 15).

### TkGGR1 shows geranylgeranyl-reducing activity and promotes TkGGPS6 activity in *N. benthamiana* leaves

**Fig. 5:**
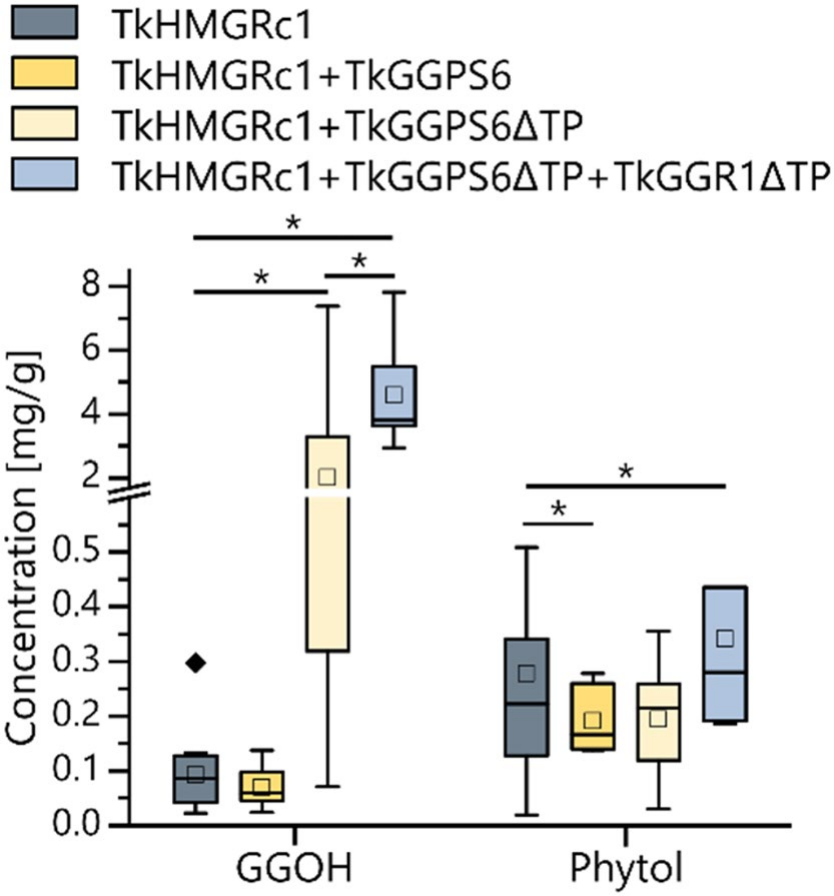
TkGGPS6/TkGGR1 co-expression in *N.* *benthamiana* increases geranylgeraniol (GGOH) and phytol levels when the transit peptides (TP) are omitted and the MVA pathway is enhanced. Quantification of phytol and GGOH in leaves of *N. benthamiana* transiently expressing the catalytic domain of *T.* *koksaghyz* 3-hydroxy-3-methylglutaryl-CoA reductase 1 (HMGR1c) (Pütter et al. 2019), TkGGPS6(∆TP) and TkGGR1∆TP. Ethyl acetate extracts were quantified by GC–MS. Box plots show values representing 18 leaves from six plants (TkHMGRc1, TkHMGRc1 + TkGGPS6∆TP) or six leaves from two plants (TkHMGRc1 + TkGGPS6, TkHMGRc1 + TkGGPS6∆TP + TkGGR1∆TP). Statistical significance was calculated using the Wilcoxon signed ranks test (*p < 0.05)

Having observed the effects of altered *TkGGR1* expression in latex on root tocopherol and triterpenoid levels, we sought to confirm the geranylgeranyl-reducing activity of TkGGR1 in leaf cells. First, we searched internal RNA-Seq data for a GGPS (providing the substrate for GGR) that is also expressed in *T.* *koksaghyz* latex and identified a gene we designated *TkGGPS6* to be consistent with recently published nomenclature (Wang et al. 2024). We then co-expressed *TkGGPS6* with *TkGGR1* in *N.* *benthamiana* leaf epidermal cells and quantified GGOH and phytol by GC–MS. To increase the IPP substrate pool, we expressed the catalytic domain of *T.* *koksaghyz* 3-hydroxy-3-methylglutaryl-CoA reductase 1 (TkHMGR1c), the rate-limiting enzyme of the cytoplasmic MVA pathway (Pütter et al. 2019), together with TkGGR1 and TkGGPS6. *TkGGPS6/TkHMGRc1* co-expression did not increase the content of GGOH, the alcohol derivative of GGPP, compared to *TkHMGRc1* expression alone (Fig. 5a). However, the co-expression of *TkHMGRc1* and a truncated *TkGGPS6* lacking the TP sequence (*TkGGPS6∆TP*) led to a significant increase in GGOH, probably due to the retention of TkGGPS6 in the cytoplasm where MVA-derived IPP was available (Fig. 5a). This confirmed the GGPP-producing activity of the newly identified TkGGPS6. The additional expression of TkGGR1∆TP resulted in higher levels of phytol compared to control leaves expressing only TkHMGRc1, confirming the geranylgeranyl-reducing activity of the latex-specific enzyme. Further, GGOH levels increased when TkGGR1∆TP was co-expressed with TkGGPS6∆TP, suggesting that TkGGR1 promotes TkGGPS6 activity. We also observed decreased phytol levels in leaves expressing TkHMGR1c/TkGGPS6 as compared to those expressing TkHMGR1c.

### *TkGGPS6* and *TkLIL3* are additional plastid-related genes expressed in *T. koksaghyz* latex

**Fig. 6:**
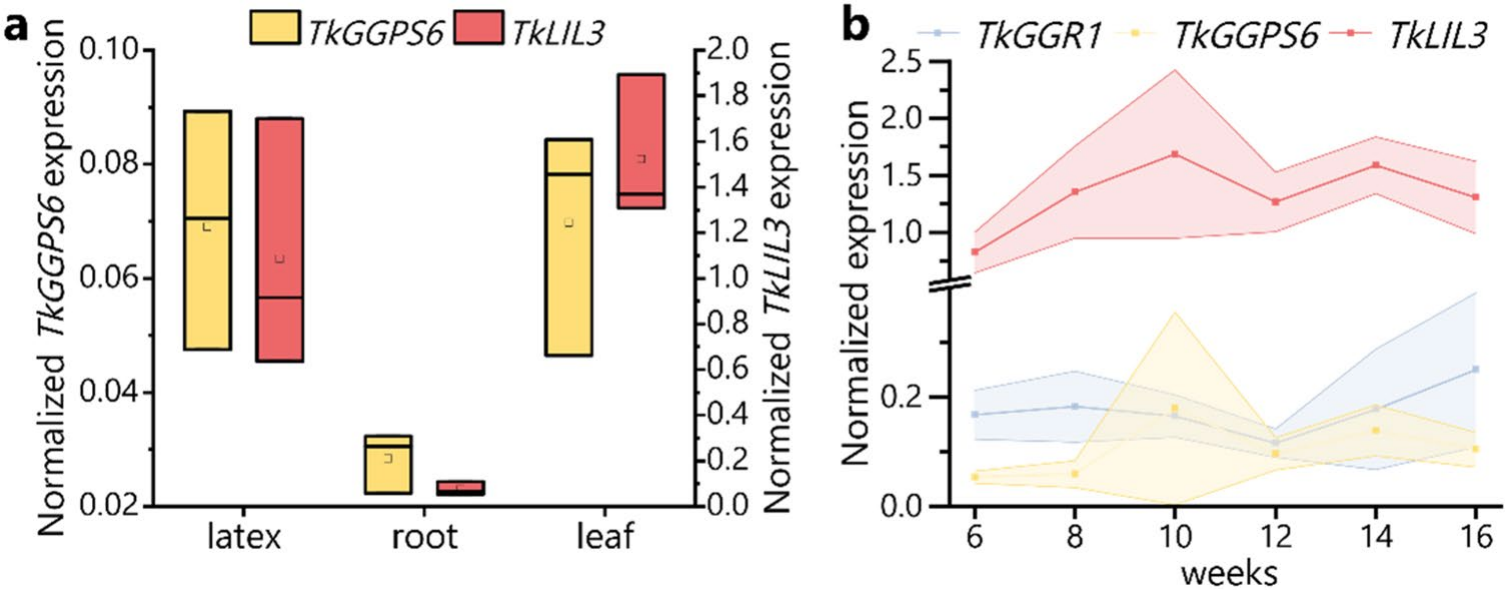
*TkGGPS6* and *TkLIL3* are consistently expressed in the latex of *T.* *koksaghyz* over time. **a** *TkGGPS6* and *TkLIL3* are expressed in *T.* *koksaghyz* latex. Normalized gene expression levels in different tissues of 12-week-old wild-type *T. koksaghyz* plants. Box plots represent values from three pools consisting of cDNA from four individual plants. **b** *TkGGR1*, *TkGGPS6* and *TkLIL3* have relatively constant expression levels in latex over time. Normalized expression levels in latex harvested at different time points. Values represent means of four individual plants per time point. The shaded areas represent the areas within the standard deviation. *TkGGR1* expression represents the sum of transcripts from both gene copies. Expression levels were normalized against *elongation factor-1 α* (*TkEF1α*) and *ribosomal protein L27* (*TkRP*)

Another enzyme that has been linked to GGRs is light-harvesting-like 3 protein (LIL3), which is thought to stabilize GGR by anchoring it to thylakoid membranes (Takahashi et al. 2014; Tanaka et al. 2010; Zhou et al. 2017). In rice, a multiprotein complex has been detected comprising GGR, a GGPS/GGPS recruiting protein (GRP) heterodimer, LIL3 and two other enzymes required for chlorophyll synthesis (Zhou et al. 2017). This raised the question as to whether a LIL3 protein may also be present in *T.* *koksaghyz* latex and may interact with TkGGR1. Interestingly, a LIL3 homolog (TkLIL3) was enriched with TkSRPP4 in our initial AE–MS experiment (Wolters et al. 2024). Our quantitative data analysis strategy suggested it was not a significantly enriched protein, because missing value imputation introduced high variance between replicates. However, this does not exclude a genuine interaction and the finding is particularly interesting given the information presented about TkGGR1. Protein domain prediction indicated one light-harvesting complex (LHC) motif and two transmembrane domains in TkLIL3, as found in AtLIL3, which further supports its identity (Supplementary Fig. 10) (Tanaka et al. 2010). Analysis of *TkLIL3* and *TkGGPS6* expression in different tissues of wild-type *T.* *koksaghyz* plants by qPCR revealed surprisingly high levels of *TkLIL3* mRNA in latex and also confirmed *TkGGPS6* expression in latex, supporting our internal RNA-Seq data (Fig. 6a). *TkLIL3* was also strongly expressed in leaves, as assumed for its chloroplast-localized homologs (Takahashi et al. 2014; F. Zhou et al. 2017). A time course experiment over 6–16 weeks, monitoring gene expression in latex, showed comparable *TkGGR1* expression over time with a slight increase from 12 to 16 weeks, whereas *TkLIL3* and *TkGGPS6* showed a generally increasing expression trend between 6 and 14 weeks (Fig. 6b). It was thus established that two genes known to be associated with GGR are expressed concurrently with *TkGGR1* in latex.

### *TkGGR1*,* TkGGPS6* and *TkLIL3* are localized at distinct sites within the chloroplast

**Fig. 7:**
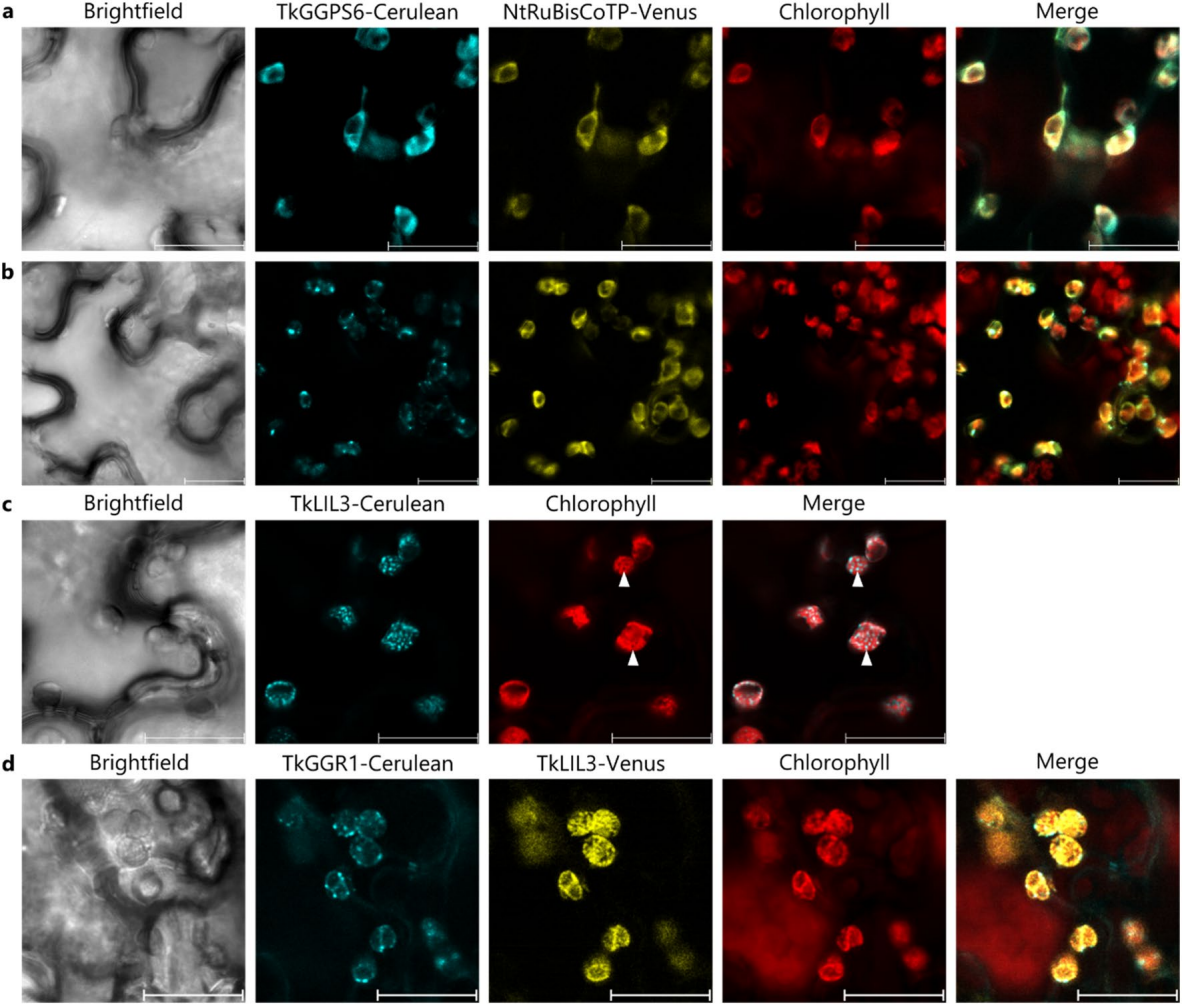
*TkGGPS6* and *TkLIL3* are imported into chloroplasts in *N.* *benthamiana*. *N. benthamiana* leaf epidermal cells expressing C-terminal Cerulean (cyan) or Venus fusion constructs (yellow). Chlorophyll autofluorescence is shown in red. a. TkGGPS6–Cerulean fluorescence overlaps with NtRuBisCO-TP-Venus (marking the chloroplast stroma). b. TkGGPS6–Cerulean fluorescence is punctuate and differs from NtRuBisCO-TP-Venus fluorescence within chloroplasts that are marked by chlorophyll autofluorescence. c. TkLIL3–Cerulean fluorescence appears as numerous spots within the area of chloroplasts marked by chlorophyll autofluorescence. d. TkLIL3-Venus fluorescence is widespread and spotty within the chloroplasts and partially overlaps the punctuate TkGGR1–Cerulean signals. Scale bars = 20 µm

Given that *TkGGPS6* and *TkLIL3* are expressed in latex, we determined whether the corresponding proteins are imported into chloroplasts in *N. benthamiana,* as observed for TkGGR1. We therefore expressed C-terminal Cerulean fusions in *N.* *benthamiana* leaf epidermal cells. The TkGGPS6–Cerulean signal partially overlapped with chlorophyll autofluorescence and exhibited two distinct patterns. In some cells, the signal was evenly distributed within the chloroplasts and largely coincided with the signal of the co-expressed stromal marker, the N-terminal TP of tobacco RuBisCO C-terminally fused to Venus (Fig. 7a). In other cells, the Cerulean signal appeared more spotted, along with some background fluorescence within the chloroplasts, which suggested localization to the thylakoids or plastoglobuli similar to TkGGR1 (Fig. 7b). TkLIL3–Cerulean fluorescence occurred as widespread points inside the chloroplasts, but the pattern differed from that of TkGGR1 and TkGGPS6 in terms of the number and arrangement of the fluorescent spots (Fig. 7c). Furthermore, image overlays indicated that chlorophyll fluorescence was absent from at least some of these punctuate structures (arrowheads Fig. 7c) suggesting they were plastoglobuli rather than thylakoids (which contain chlorophyll). The co-expression of TkLIL3-Venus with TkGGR1–Cerulean further suggested that TkLIL3 and TkGGR1 localization inside chloroplasts partially overlapped at plastoglobuli and/or thylakoids but was not identical (Fig. 7d).

### TkGGR1 interacts with latex-abundant TkGGPS6 and TkSRPP4-interacting TkLIL3

**Fig. 8:**
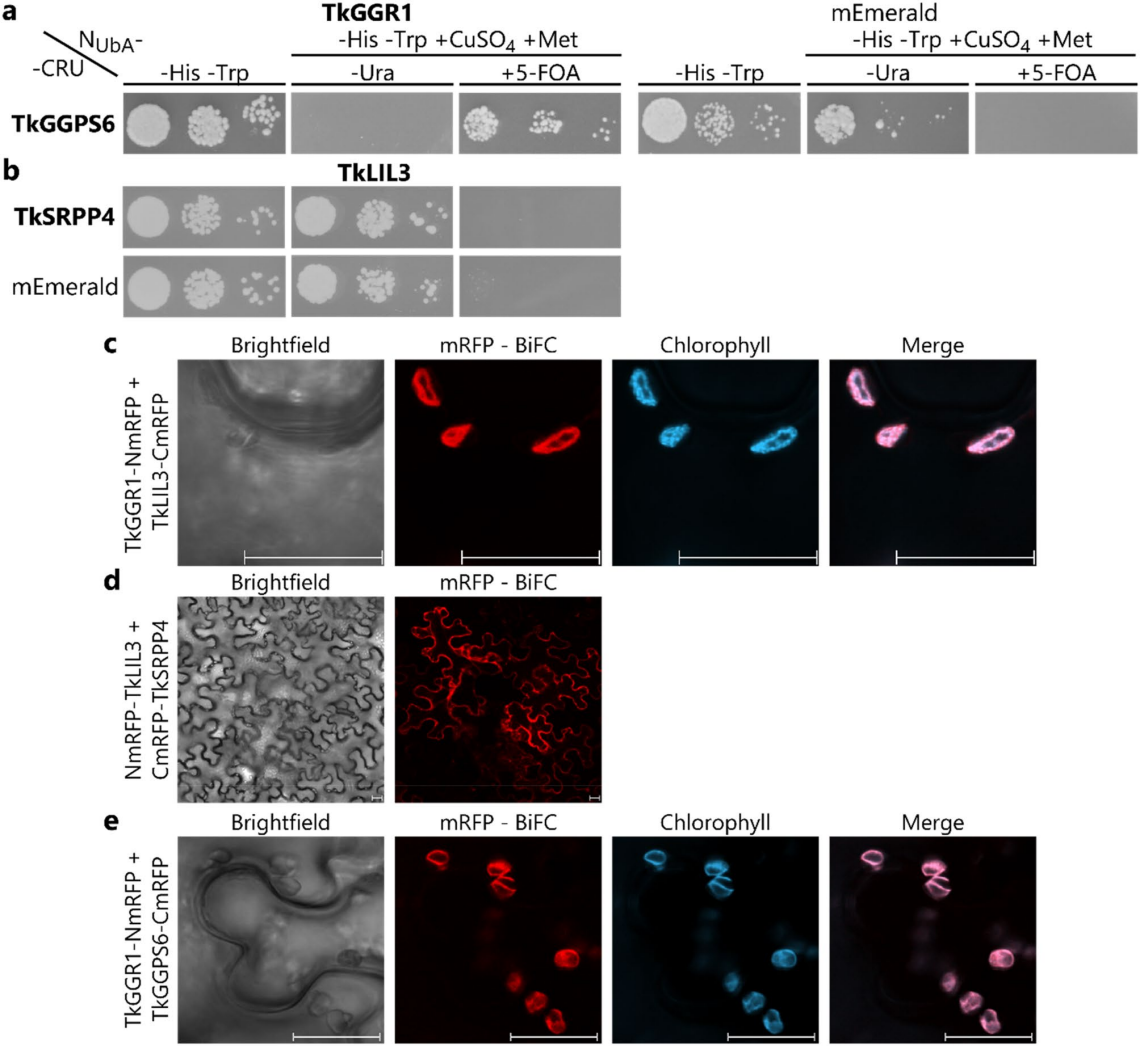
TkGGR1 interacts with TkGGPS6 and TkLIL3 inside chloroplasts whereas TkLIL3 interacts with TkSRPP4 externally. **a** Split-ubiquitin membrane yeast-two hybrid (SUY2H) assay indicating an interaction between TkGGR1 and TkGGPS6. **b** SUY2H rejecting the TkLIL3/TkSRPP4 interaction. Yeast expressing TkGGR1 or TkLIL3 N-terminally fused to the N-terminal part of ubiquitin (N_UbA_) and TkGGPS6 or TkSRPP4 C-terminally fused to the ubiquitin C-terminus and the URA3 reporter (CRU) were dropped in three different dilutions on selective media and grown for 2–3 days. Medium lacking histidine and tryptophan (–H–T) was used as a control only selecting for the plasmids encoding both fusion proteins. Medium additionally lacking uracil and containing 50 µM CuSO_4_ and 300 µM methionine (–H–T–U + CuSO_4_ + M) was used to select for URA3 activity. Medium containing uracil and 1 g/L 5-FOA (–H–T + CuSO_4_ + M + 5-FOA) was used to select for URA3 inactivity, confirming protein interactions. The fluorophore mEmerald was used as a negative control. **c**, **d**, **e** Bimolecular fluorescence complementation (BiFC) in *N.* *benthamiana*. Candidate proteins fused in both orientations to the N-terminal or C-terminal part of monomeric red fluorescent protein (NmRFP or CmRFP) were transiently co-expressed in *N.* *benthamiana* leaf epidermal cells. Chlorophyll autofluorescence is shown in blue (false color) to mark the chloroplasts. c. TkGGR1/TkGGPS6 interaction indicated by red mRFP fluorescence inside chloroplasts d. TkLIL3/TkSRPP4 interaction indicated by mRFP fluorescence outside the chloroplasts. e. TkGGR1/TkGGPS6 interaction indicated inside chloroplasts. Scale bars = 20 µm (Colour figure online)

Given that TkGGPS6 and TkLIL3, like TkGGR1, are imported into chloroplasts in leaf cells, and that interactions between GGR, GGPS and LIL3 have been described in other species (Ruiz-Sola et al. 2016a, b; Zhou et al. 2017), we determined whether TkGGR1 also interacts with these proteins using the SUY2H system. This indicated protein interaction between TkGGR1 and TkGGPS6 (Fig. 8a), but TkLIL3/TkSRPP4 interaction initially suggested by AE–MS (Wolters et al. 2024) was not confirmed using this system (Fig. 8b). TkGGR1/TkLIL3 interaction could not be tested due to false positive interactions with the negative control mEmerald, when either TkGGR1 or TkLIL3 was C-terminally fused to CRU (data not shown). We therefore used BiFC to assess the TkGGR1/TkLIL3 and TkLIL3/TkSRPP4 interactions in planta. The candidate interacting proteins were fused to NmRFP or CmRFP and co-expressed in *N. benthamiana*. Co-expression with the mEmerald fluorophore fused to NmRFP or CmRFP, respectively, served as a negative control (Supplementary Fig. 16). The co-expression of TkGGR1-NmRFP and TkLIL3-CmRFP showed TkGGR1/TkLIL3 interaction inside chloroplasts (Fig. 8c). TkLIL3/TkSRPP4 interaction was observed by BiFC only when TkLIL3 was N-terminally fused to NmRFP and due to the shielding of the TP not translocated to the chloroplasts (Fig. 8d). The intracellular site of the detected interaction cannot be clearly deduced from our microscopic images. Based on the affinity of TkLIL3 to membranes within the chloroplasts indicated by our localization analysis (Fig. 7c), and ER localization of the closely related TbSRPP4 (Laibach et al. 2015a, b), a TkLIL3/TkSRPP4 interaction at the ER might be most likely. Furthermore, BiFC analysis also confirmed the TkGGR1/TkGGPS6 interaction, which was also located inside chloroplasts (Fig. 8e).

## Discussion

The *T. koksaghyz* genome encodes multiple GGRs and we have identified one paralog, designated TkGGR2, which appears to be the main enzyme contributing to the full reduction of the chlorophyll side chain in photosynthetically active tissues. Similar to the silencing of *N. tabacum CHLP*, *TbGGR2* silencing resulted in increased amounts of chl_GG_ (Tanaka et al. 1999). But in contrast to tobacco, *TbGGR2*-RNAi plants tended to contain higher instead of lower chlorophyll levels when compared with wild-type plants, a difference that might be associated with the phylogenetic divergence of both protein (Fig. 1b). Besides chlorophyll, TkGGR2 also probably contributes to tocopherol synthesis in chloroplasts and thereby helps to maintain the antioxidant potential of cells, as described for homologs in other species (Graßes et al. 2001; Kimura et al. 2018). It would be interesting to test whether *GGR2*-RNAi plants are more sensitive to light stress, as described for *NtCHLP* antisense plants (Graßes et al. 2001). *TkGGR3* was differentially expressed in leaves overexpressing *TkPEL*-*like* (Wolters et al. 2023) but *TkGGR3* mRNA was not detected by qPCR in wild-type *T.* *koksaghyz* leaves, roots or latex. Therefore, TkGGR3 is unlikely to have a primary role in photosynthesis-related PhyPP supply and it may be a functionally specialized paralog expressed in response to certain stimuli. This is supported by the transcriptional upregulation of a gene homologous to *AtCHLP* in *T.* *koksaghyz* roots following treatment with methyl jasmonate (Cao et al. 2017). A similar specialization could explain the presence of TkGGR1, which is mainly expressed in latex together with its plastid-localized interactors TkLIL3 and TkGGPS6. The function of GGR in chlorophyll synthesis appears to be fulfilled by TkGGR2, and the expression of *TkGGR1* in latex gave the first indication that it has a specialized function in the isoprenoid network of this tissue, providing new insights into latex biology.

Metabolic analysis in *N.* *benthamiana* leaves transiently expressing TkHMGRc1, TkGGPS6∆TP and TkGGR1∆TP (Fig. 5) indicated that the PhyPP-producing activity of TkGGR1 is conserved. This was supported by the lower tocopherol levels in the roots of *T.* *koksaghyz TkGGR1*-RNAi plants compared to wild-type controls (Fig. 4b) and the upregulation of *TbGGR1* in *T.* *brevicorniculatum TbGGR2*-RNAi plants with dark green leaves, which contained more chl_Phy_ than the pale green plants of the same genotype (Fig. 2d, g, h). Interestingly, a negative effect on tocopherols in roots was also observed following the upregulation of *TkGGR1* in *T.* *koksaghyz* latex (Fig. 4b). This could be explained by a hypothesis proposed earlier for chloroplasts and seeds where phytol and PhyPP directly derived from GGR may not be accessible for tocopherol synthesis, which only utilizes PhyPP derived from phytol phosphorylation (Lippold et al. 2012; Valentin et al. 2006; vom Dorp et al. 2015). Enhanced TkGGR1 activity may led to higher phytol levels, which could have promoted free fatty acid esterification (and thereby detoxification). The increasingly esterified phytol thus may have been unavailable for phosphorylation and subsequent incorporation into tocopherols. The presumption that tocopherol is produced in latex is supported by the detection of a homogentisate phytylprenyltransferase, key enzyme in tocopherol synthesis, in *T.* *koksaghyz* latex (Niephaus et al. 2019) and α-tocopherol and tocotrienols, which are based on GGPP instead of PhyPP, in the latex of *Hevea brasiliensis* (Dunphy et al. 1965). Metabolic analysis of the roots of *GGR1*-RNAi and P_REF_-*TkGGR1* plants further indicated that GGR1 does not generally interfere with the production of the triterpenoid precursor FPP, or IPP used for NR chain elongation (Fig. 4, Supplementary Figs. 11, 12). Accordingly, no direct link could be established between the different NR contents and *GGR1*/*GGR2* expression levels in the latex of *T.* *koksaghyz* and *T.* *brevicorniculatum* plants. We were also unable to draw a link between the function of GGR1 and the different effects on individual triterpenoids and sterols in the roots of each dandelion species. However, these effects provide evidence for the activity of GGR1 in the highly branched isoprenoid network in latex. We hypothesize that TkGGR1 catalyzes PhyPP synthesis from GGPP or phytol synthesis from GGOH, which could be used for tocopherol synthesis and the esterification/detoxification of free fatty acids, which are products of oxidative stress. These fatty acid phytyl esters (FAPEs) accumulate as storage fats inside chloroplast plastoglobuli (Gaude et al. 2007) and could also be stored inside rubber particles or plastoglobuli derivatives in latex. To prove this hypothesis, future work should focus on the analysis of the *T.* *koksaghyz TkGGR1*-RNAi and P_REF_-*TkGGR1* plants under oxidative stress conditions.

Although the precise physiological role of TkGGR1 in the latex isoprenoid network was not elucidated by our experiments, the identification of TkGGR1 and TkLIL3 mRNA and protein (Wolters et al. 2024) in *T.* *koksaghyz* latex provided new information about latex physiology. Their presence in latex despite their import into *N.* *benthamiana* leaf chloroplasts raised questions about their localization in latex, which lacks genuine chloroplasts. The analysis of transgenic plants indicated that TkGGR1-mVenus is imported into plastid-like particles that accumulate in the latex pellet phase after centrifugation (Fig. 3c, d), supported by the enrichment of TkGGR1 with TkSRPP3 from the pellet phase in our AE–MS experiments (Wolters et al. 2024). Notably, plastid-associated proteins were also dominant in *L. sativa* latex and included a GGR homolog (Cho et al. 2009). The observed structures are similar to Frey–Wyssling (F.W.) complexes, which are specialized plastids present in the latex of the rubber tree *H. brasiliensis* (Frey-Wyssling 1929). Ultrastructural analysis showed a range of morphologies for these organelles, but they had in common a delimiting double membrane, lipid globules, vesicles and a ‘gray body’ in association with membrane configurations (Gomez and Hamzah 1989; Moir 1959). They are 3–7 µm in diameter and yellowish-orange in color, most likely caused by the presence of carotenoids (Dickenson 1965; Gomez and Hamzah 1989). Given the complexity of these structures, the authors suggested they are unlikely to be exclusive to the genus *Hevea* (Dickenson 1969), and plastid-like structures were later also observed in *T. koksaghyz* latex and possibly in a separate latex phase after centrifugation (Ghaffar 2017; Liu et al. 2024). This aligns with the identification of a latex-specific polyphenol oxidase (PPO) with a functional chloroplast TP in *T.* *brevicorniculatum*, which led to the hypothesis that F.W. complexes are present in dandelion latex and contain TbPPO (Wahler et al. 2009). We propose that, like TkGGR1, TkLIL3 and TkGGPS6 are also imported into F.W.-like complexes in latex. The generation and analysis of plants expressing fluorophore fusions under the control of the strong latex-specific P_REF_, analogous to those presented for TkGGR1 here (Fig. 3), will help to confirm their precise cellular localization. The affinity of TkGGR1 for plastid and LD membranes suggests an association with lipid globules, probably derived from plastoglobuli or rubber particles inside F.W-like complexes. This is supported by the detection of high-density NR inside latex plastids by electron microscopy (Dickenson 1969; Ghaffar and Cornish 2020) and the identification of TkGGR1 homologs in *T.* *brevicorniculatum* rubber particles (Wahler et al. 2012). However, TkGGR1∆TP did not associate with LDs in the cytosol of *N.* *benthamiana* (Supplementary Fig. 13c), indicating that TkGGR1 has an affinity for specific lipids and a particular membrane composition and/or that interaction with other proteins may be essential for the binding of TkGGR1.

The pairwise interactions of TkGGR1 with TkLIL3 and TkGGPS6 suggest the formation of a tripartite complex within F.W.-like complexes. In Arabidopsis leaves, LIL3 is located on thylakoid membranes (Takahashi et al. 2014), which is consistent with our results that suggest the localization of TkLIL3 on thylakoid membranes and in plastoglobuli. However, the precise location of TkLIL3 within F.W.-like complexes cannot be deduced because little information is available about the architecture of these complexes. TkLIL3 may stabilize TkGGR1 in a membrane-associated complex (Tanaka et al. 2010) and its LHC motif might interact with TkGGR1 to promote the membrane anchoring of TkGGR1, which is essential for GGR activity in Arabidopsis (Takahashi et al. 2014). Given that TkGGR1 interacts with TkLIL3 inside chloroplasts (Fig. 8c), but both proteins do not fully co-localize under our experimental conditions (Fig. 7d), substantial protein dynamics are likely inside the chloroplasts as already indicated by the dynamic association of plastoglobuli with thylakoid membranes (van Wijk and Kessler 2017). The identification of a TkLIL3 homolog in the rubber particle proteome of *T.* *brevicorniculatum* suggests TkLIL3 is localized on the surface of rubber particles, where it could stabilize TkGGR1 (Wahler et al. 2012). This interaction may also involve substrate or product shuttling by TkLIL3 to facilitate metabolic channeling and prevent oxidative damage by pathway intermediates.

Transcriptomic data from a recent study focusing on the *TkGGPS* gene family confirmed *TkGGPS6* expression in latex and indicated that another paralog (*TkGGPS3*) is also strongly expressed in this tissue (Wang et al. 2024). Based on these findings, we can envisage similar or diverging functions for these proteins in latex. In contrast to the aforementioned study, we were able to amplify the full-length *TkGGPS6* coding sequence from genomic DNA, indicating the absence of introns in this gene (data not shown). In our experiments, TkGGPS6 showed two different localization patterns in *N.* *benthamiana* chloroplasts (Fig. 7a, b), one largely matching the previous report (Wang et al. 2024). Such patterns were similarly observed for OsGGPS1, which is located in the stroma as a homodimer but is recruited to the thylakoids when it forms a heterodimer with OsGRP (F. Zhou et al. 2017). TkGGPS6 therefore may have formed heterodimers with a *N.* *benthamiana* GRP homolog leading to its recruitment to the thylakoids and/or plastoglobuli, but was distributed in the stroma in the absence of a partner. The *T.* *koksaghyz* latex proteome contains a protein sharing 53% identity with OsGRP and 64% with a heterodimeric GGPS small subunit from Arabidopsis (Niephaus et al. 2019) that was also identified in the *T.* *brevicorniculatum* rubber particle proteome (Wahler et al. 2012). Consequently, this protein is a candidate interaction partner to form heterodimers with TkGGPS6 in latex, which might modulate its localization and thereby its participation in different metabolic pathways. GGPS small subunits were also shown to change GGPS product specificity or enhance both specificity and activity (Wang and Dixon 2009; Zhou and Pichersky 2020), the latter of which we have also observed for TkGGR1 in *N.* *benthamiana*. Within F.W.-like complexes, the proposed localization of TkGGPS6 in latex, it might be similarly found in an aqueous stroma-like compartment or associated with membranes, potentially in a complex with a TkGRP. TkGGPS6 may also be recruited to rubber particles by interaction with TkGGR1 or TkGRP, and this is supported by the identification of a GGPS on the surface of *H.* *brasiliensis* rubber particles (Wang et al. 2019). The suggested presence of TkGGPS6 in F.W.-like complexes in *T. koksaghyz* latex is supported by a GGPP-synthesizing activity in bottom fractions of *H.* *brasiliensis* latex (Tangpakdee et al. 1997). Furthermore, the yellowish-orange color of F.W. complexes in *H.* *brasiliensis* indicates the storage of carotenoids, which require GGPP for their synthesis (Chow et al. 2012; Moir 1959). We occasionally observed orange coloring at the very bottom of the *T.* *koksaghyz* pellet phase after centrifugation, which similarly hints at the presence of carotenoids that could be TkGGPS6 end-products in latex, or more specifically F.W.-like complexes (Supplementary Fig. 17). The product of TkGGPS6 (GGPP) may also be involved in protein prenylation, which can modify a protein’s function and localization by increasing its hydrophobicity (Turnbull and Hemsley 2017). The post-translational modification of latex proteins may also be mediated by TkGGR1, given that an unconventional type of phytol-prenylation that is limited to chloroplasts has been identified in spinach (Parmryd et al. 1999). The allylic diphosphate starter molecule for NR polymerization has yet to be clearly identified, so it is also possible that TkGGPS6 contributes to the supply of this starter molecule. This is supported by the fact that two TkGGPS paralogs were significantly enriched in the latex of *Tkcpt1/2* knockout plants lacking key components of the NR-producing enzyme complex (Xu et al. 2025).

Initially, TkGGR1 caught our attention because it interacted with TkSRPP3 (Wolters et al. 2024), but the precise site of interaction within latex has yet to be determined. Thus far, SRPPs have only been described in rubber particles within latex, and unlike TkGGR1, TkLIL3 and TkGGPS6, they do not contain a chloroplast TP, which makes it unlikely that they are imported into F.W.-like complexes. In agreement with this, TkLIL3/TkSRPP4 interactions were only detected by BiFC when TkLIL3 chloroplast import was prevented by N-terminal fluorophore fusion (Fig. 8d). The interaction between TkGGR1 and TkSRPP3 could not be verified by BiFC because TkSRPP3 interacted with the control fluorophore, but the closely related TbSRPP3 and TbSRPP4 were not imported into chloroplasts when expressed in *N. benthamiana* (Laibach et al. 2018). Nevertheless, TkSRPP3 interaction partners in latex identified by AE–MS were significantly enriched for Gene Ontology (GO) terms related to chloroplasts and thylakoids, demonstrating a clear link between TkSRPP3 and plastids (Wolters et al. 2024). Electron microscopy images indicated that ER-derived vesicles or the ER directly released small rubber particles into the laticifer plastid lumen and that small rubber particles are directly translocated into the plastid from the cytosol (Ghaffar and Cornish 2020). Information on plastidial rubber particles is currently very limited, so assumptions made about their characteristics must therefore be considered with caution. However, based on the microscopic observations (Ghaffar and Cornish 2020), interactions between TkSRPPs, TkGGR1 and TkLIL3 could occur inside the F.W.-like particles following the intake of TkSRPP-coated rubber particles. In this scenario, TkGGR1 and TkLIL3 could be recruited to the imported rubber particles by TkSRPPs or TkGGR1 could associate with the rubber particles through its intrinsic affinity to LDs and then interact with TkSRPP3 there. Given that TkGGR1 is thought to differ in affinity for LDs with certain phospholipid compositions, TkSRPP binding could also promote or modulate the association of TkGGR1 and TkLIL3 with rubber particle membranes. The ability of TkGGR1 and TkLIL3 to associate with rubber particles is reinforced by the identification of *T.* *brevicorniculatum* homologs in the rubber particle proteome (Wahler et al. 2012) and a weak band representing TkGGR1-mVenus in the rubber phase (Fig. 3c). Similar to vacuolar plastoglobule degradation following extrusion from chloroplasts undergoing degradation (Domínguez and Cejudo 2021), transmission electron microscopy indicated the translocation of plastidial and also cytoplasmic rubber particles into the vacuole (Ghaffar and Cornish 2020). Therefore, the proteins could also make contact on the surface of rubber particles or plastoglobuli-like LDs inside the vacuole. Another possible mechanism for TkGGR1/TkSRPP3 and TkLIL3/TkSRPP4 interactions could be the release of the plastid proteins from F.W.-like complexes through complex dissipation into the cytosol under certain stress conditions or during aging, similar to chloroplast-to-gerontoplast development (Domínguez and Cejudo 2021). In this manner, TkGGR1 and TkLIL3 could interact with TkSRPPs on cytoplasmic rubber particles or in compartments such as the endoplasmic reticulum (ER). ER localization is supported by the presence of TbSRPP3 and TbSRPP4 in the ER of *N.* *benthamiana* (Laibach et al. 2018), the TkLIL3/TkSRPP4 interaction indicated by BiFC (Fig. 8a), the enrichment of TkGGR1 with TkSRPP3 from the pellet phase in AE–MS experiments, and the detection of TkSRPP3 and TkSRPP4 in the pellet phase (Wolters et al. 2024). F.W.-like complex dissipation could also enable TkSRPPs to associate with released plastoglobuli derivatives and their postulated surface proteins TkGGR1 and TkLIL3 in the cytosol. Regarding the potential interaction between TkGGR1 and TkSRPP3 in the latex, it must be considered that (based on the mRNA level) TkGGR1 protein abundance appears to be rather low whereas TkSRPP3 levels are extremely high (Wolters et al. 2024; Niephaus et al. 2019). This makes the analysis of the proteins’ binding affinities and their binding ratio an important aspect for future studies.

The TkGGR1/TkLIL3 interaction most likely stabilizes TkGGR1 on membranes, resulting in an adequate supply of hydrophobic substrates. The TkGGR1/TkGGPS6 interaction probably achieves metabolite channeling and enhances TkGGPS6 activity, as indicated by co-expression in *N.* *benthamiana* (Fig. 5). The purpose of TkSRPP3 and TkSRPP4 interactions with TkGGR1 and TkLIL3 is unclear, and additional studies are needed. However, TkSRPP3 and TkSRPP4 may expand the proposed TkGGR1/TkLIL3/TkGGPS6 complex as structural components and may influence its cellular location based on distinct lipid affinities. Interaction and relocation could be triggered by external stimuli given that *SRPP* expression is stress-responsive, TkSRPP3 and TkSRPP4 both harbor phosphorylation and *Ν*-glycosylation acceptor sites (Guo et al. 2014; Laibach et al. 2018; Seo et al. 2010; Wolters et al. 2024), and a *AtCHLP* homolog was induced in *T.* *koksaghyz* roots after methyl jasmonate treatment (Cao et al. 2017). Relocation could be part of a TkSRPP-mediated downstream process to counter stress given that SRPPs, GGR and LIL3 have been positively correlated with stress tolerance (Kim et al. 2016; Laibach et al. 2018; Zhou et al. 2013). The recruitment of the complex containing TkGGR1 could redirect the metabolic flux associated with TkGGR1/TkGGPS6 activity. A possible scenario is TkSRPP-directed PhyPP synthesis for the esterification/detoxification of free fatty acids as proposed above. The resulting FAPEs could be stored directly inside rubber particles or derivatives of plastoglobuli. This is corroborated by the lipid-rich environment of latex, in which high levels of lipid peroxidation are likely to occur. TkSRPP interaction with TkGGR1 and TkLIL3 may therefore be a mechanism by which TkSRPPs confer tolerance to oxidative stress. Further, TkGGR1/TkSRPP3 interaction could play a role in rubber particle stabilization because tocopherols, potential downstream products of TkGGR1, have been shown to affect the architecture and oxidative stability of lipid droplets and solid lipid nanoparticles (Olbińska et al. 2023; Stahl et al. 2024).

The conservation of proteins required for photosynthesis-associated chlorophyll and tocopherol synthesis in specialized cells such as latex-containing laticifers, which are barely exposed to light and do not contain conventional chloroplasts, is remarkable and broadens our knowledge of the isoprenoid network in latex. Our findings provide insight into the complexity of latex metabolism and physiology, and will contribute to a more detailed understanding of this tissue, thus facilitating its further exploitation for the benefit of humans.

## Supplementary Information

Below is the link to the electronic supplementary material.

Supplementary file1 (DOCX 6140 KB)

## Data Availability

The gene sequences are available at the gene banks listed in the Materials and Methods section and Supplementary Table 2, each with its corresponding accession number.

## References

[CR1] Agatep R, Kirkpatrick RD, Parchaliuk DL, Woods RA, Gietz RD (1998) Transformation of Saccharomyces cerevisiae by the lithium acetate/single-stranded carrier DNA/polyethylene glycol protocol. Techn Tips Online 3(1):133–137. 10.1016/s1366-2120(08)70121-1

[CR2] Alberti S, Gitler AD, Lindquist S (2007) A suite of Gateway ® cloning vectors for high-throughput genetic analysis in *Saccharomyces cerevisiae*. Yeast 24(10):913–919. 10.1002/yea.1502 17583893 10.1002/yea.1502 PMC2190539

[CR3] Beck G, Coman D, Herren E, Ruiz-Sola MÁ, Rodríguez-Concepción M, Gruissem W, Vranová E (2013) Characterization of the GGPP synthase gene family in Arabidopsis thaliana. Plant Mol Biol 82(4–5):393–416. 10.1007/s11103-013-0070-z 23729351 10.1007/s11103-013-0070-z

[CR4] Benninghaus VA, Van Deenen N, Müller B, Roelfs KU, Lassowskat I, Finkemeier I, Prüfer D, Gronover CS (2020) Comparative proteome and metabolome analyses of latex-exuding and non-exuding Taraxacum koksaghyz roots provide insights into laticifer biology. J Exp Bot 71(4):1278. 10.1093/JXB/ERZ512 31740929 10.1093/jxb/erz512 PMC7031084

[CR5] Böttner L, Malacrinò A, Schulze Gronover C, van Deenen N, Müller B, Xu S, Gershenzon J, Prüfer D, Huber M (2023) Natural rubber reduces herbivory and alters the microbiome below ground. New Phytol. 10.1111/NPH.18709 36597727 10.1111/nph.18709

[CR6] Bruno L, Chiappetta A, Muzzalupo I, Gagliardi C, Iaria D, Bruno A, Greco M, Giannino D, Perri E, Bitonti MB (2009) Role of geranylgeranyl reductase gene in organ development and stress response in olive (Olea europaea) plants. Funct Plant Biol 36(4):370–381. 10.1071/FP08219 32688654 10.1071/FP08219

[CR7] Cao XW, Yan J, Lei JL, Li J, Zhu JB, Zhang HY (2017) De novo transcriptome sequencing of MeJA-induced Taraxacum koksaghyz Rodin to identify genes related to rubber formation. Sci Rep 7(1):1–13. 10.1038/s41598-017-14890-z 29146946 10.1038/s41598-017-14890-z PMC5691164

[CR8] Cho WK, Chen XY, Uddin NM, Rim Y, Moon J, Jung JH, Shi C, Chu H, Kim S, Kim SW, Park ZY, Kim JY (2009) Comprehensive proteome analysis of lettuce latex using multidimensional protein-identification technology. Phytochemistry 70(5):570–578. 10.1016/J.PHYTOCHEM.2009.03.004 19356777 10.1016/j.phytochem.2009.03.004

[CR9] Chow K-S, Mat-Isa M-N, Bahari A, Ghazali A-K, Alias H, Mohd.-Zainuddin Z, Hoh C-C, Wan K-L (2012) Metabolic routes affecting rubber biosynthesis in Hevea brasiliensis latex. J Exp Bot 63(5):1863–1871. 10.1093/jxb/err363 22162870 10.1093/jxb/err363 PMC3295384

[CR10] Collins-Silva J, Nural AT, Skaggs A, Scott D, Hathwaik U, Woolsey R, Schegg K, McMahan C, Whalen M, Cornish K, Shintani D (2012) Altered levels of the Taraxacum kok-saghyz (Russian dandelion) small rubber particle protein, TkSRPP3, result in qualitative and quantitative changes in rubber metabolism. Phytochemistry 79:46–56. 10.1016/j.phytochem.2012.04.015 22609069 10.1016/j.phytochem.2012.04.015

[CR11] Conart C, Bomzan DP, Huang XQ, Bassard JE, Paramita SN, Saint-Marcoux D, Rius-Bony A, Hivert G, Anchisi A, Schaller H, Hamama L, Magnard JL, Lipko A, Swiezewska E, Jame P, Riveill G, Oyant LHS, Rohmer M, Lewinsohn E, Boachon B (2023) A cytosolic bifunctional geranyl/farnesyl diphosphate synthase provides MVA-derived GPP for geraniol biosynthesis in rose flowers. Proc Natl Acad Sci U S A. 10.1073/PNAS.2221440120 37126706 10.1073/pnas.2221440120 PMC10175749

[CR12] Dickenson PB (1965) The ultrastructure of the latex vessel of Hevea brasiliensis. In: Mullins L (ed) Proceedings of the natural rubber producers research association jubilee conference. Mclaren & Sons Ltd, Surrey, p 52

[CR13] Dickenson PB (1969) Electron microscopial studies of latex vessel system of Hevea brasiliensis. J Rubber Res 21:543–559

[CR14] Domínguez F, Cejudo FJ (2021) Chloroplast dismantling in leaf senescence. J Exp Bot 72(16):5905–5918. 10.1093/JXB/ERAB200 33959761 10.1093/jxb/erab200 PMC8760853

[CR15] Dunphy PJ, Whittle KJ, Pennock JF, Morton RA (1965) Identification and estimation of tocotrienols in hevea latex. Nature 207(4996):521–522. 10.1038/207521a0

[CR16] Epping J, van Deenen N, Niephaus E, Stolze A, Fricke J, Huber C, Eisenreich W, Twyman RM, Prüfer D, Schulze Gronover C (2015) A rubber transferase activator is necessary for natural rubber biosynthesis in dandelion. Nat Plants. 10.1038/nplants.2015.48

[CR17] Frey-Wyssling A (1929) Microscopic investigations on the occurrence of resins in Hevea latex. Arch Rubbercult 13:294–392

[CR18] Fricke J, Hillebrand A, Twyman RM, Prüfer D, Schulze Gronover C (2013) Abscisic acid-dependent regulation of small rubber particle protein gene expression in Taraxacum brevicorniculatum is mediated by TbbZIP1. Plant Cell Physiol 54(4):448–464. 10.1093/pcp/pcs182 23303876 10.1093/pcp/pcs182

[CR19] Gaude N, Bréhélin C, Tischendorf G, Kessler F, Dörmann P (2007) Nitrogen deficiency in Arabidopsis affects galactolipid composition and gene expression and results in accumulation of fatty acid phytyl esters. Plant J 49(4):729–739. 10.1111/j.1365-313X.2006.02992.x 17270009 10.1111/j.1365-313X.2006.02992.x

[CR20] Ghaffar MAA, Cornish K (2020) New developments in rubber particle biogenesis of rubber-producing species. In: Matsui M, Chow K-S (eds) The rubber tree genome. Springer, Cham, pp 153–168. 10.1007/978-3-030-42258-5_10

[CR21] Ghaffar MAA (2017). Rubber Particle Ontogeny in Taraxacum kok-saghyz. [Ohio State University, USA]. http://rave.ohiolink.edu/etdc/view?acc_num=osu1512031318000982

[CR22] Giannino D, Condello E, Bruno L, Testone G, Tartarini A, Cozza R, Innocenti AM, Bitonti MB, Mariotti D (2004) The gene geranylgeranyl reductase of peach (Prunus persica [L.] Batsch) is regulated during leaf development and responds differentially to distinct stress factors. J Exp Bot 55(405):2063–2073. 10.1093/jxb/erh217 15286145 10.1093/jxb/erh217

[CR23] Gomez JB, Hamzah S (1989) Frey-wyssling complex in hevea latex - uniqueness of the organelle. J Nat Prod 4(2):75–85

[CR24] Graßes T, Grimm B, Koroleva O, Jahns P (2001) Loss of α-tocopherol in tobacco plants with decreased geranylgeranyl reductase activity does not modify photosynthesis in optimal growth conditions but increases sensitivity to high-light stress. Planta 213(4):620–628. 10.1007/s004250100532 11556795 10.1007/s004250100532

[CR25] Guo D, Li HL, Tang X, Peng SQ (2014) Molecular and functional characterization of the HbSRPP promoter in response to hormones and abiotic stresses. Transgenic Res 23(2):331–340. 10.1007/S11248-013-9753-0 24043397 10.1007/s11248-013-9753-0

[CR26] He F, Shi Y-J, Chen Q, Li J-L, Niu M-X, Feng C-H, Lu M-M, Tian F-F, Zhang F, Lin T-T, Chen L-H, Liu Q, Wan X-Q (2022) Genome-wide investigation of the PtrCHLP family reveals that PtrCHLP3 actively mediates poplar growth and development by regulating photosynthesis. Front Plant Sci 13:1499. 10.3389/fpls.2022.870970 10.3389/fpls.2022.870970 PMC9127975 35620683

[CR27] He H, Wang J, Meng Z, Dijkwel PP, Du P, Shi S, Dong Y, Li H, Xie Q (2024) Genome-wide analysis of the SRPP/REF gene family in Taraxacum kok-saghyz provides insights into its expression patterns in response to ethylene and methyl jasmonate treatments. Int J Mol Sci 25(13):6864. 10.3390/IJMS25136864 38999970 10.3390/ijms25136864 PMC11241686

[CR28] Hecker A, Wallmeroth N, Peter S, Blatt MR, Harter K, Grefen C (2015) Binary 2in1 vectors improve in planta (co)localization and dynamic protein interaction studies. Plant Physiol 168(3):776–787. 10.1104/PP.15.00533 25971551 10.1104/pp.15.00533 PMC4741326

[CR29] Hillebrand A, Post JJ, Wurbs D, Wahler D, Lenders M, Krzyzanek V, Prüfer D, Schulze Gronover C (2012) Down-regulation of small rubber particle protein expression affects integrity of rubber particles and rubber content in Taraxacum brevicorniculatum. PLoS ONE 7(7):e41874. 10.1371/journal.pone.0041874 22911861 10.1371/journal.pone.0041874 PMC3402443

[CR30] Hirose M, Tsukatani Y, Harada J, Tamiaki H (2022) Characterization of regioisomeric diterpenoid tails in bacteriochlorophylls produced by geranylgeranyl reductase from Halorhodospira halochloris and Blastochloris viridis. Photosynth Res 2022(1):1–12. 10.1007/S11120-022-00938-3 10.1007/s11120-022-00938-3 35852706

[CR31] Huber M, Epping J, Schulze Gronover C, Fricke J, Aziz Z, Brillatz T, Swyers M, Köllner TG, Vogel H, Hammerbacher A, Triebwasser-Freese D, Robert CAM, Verhoeven K, Preite V, Gershenzon J, Erb M (2016) A latex metabolite benefits plant fitness under root herbivore attack. PLoS Biol 14(1):e1002332. 10.1371/journal.pbio.1002332 26731567 10.1371/journal.pbio.1002332 PMC4701418

[CR32] Jach G, Pesch M, Richter K, Frings S, Uhrig JF (2006) An improved mRFP1 adds red to bimolecular fluorescence complementation. Nat Methods 3(8):597–600. 10.1038/nmeth901 16862132 10.1038/nmeth901

[CR33] Jekat SB, Ernst AM, von Bohl A, Zielonka S, Twyman RM, Noll GA, Prüfer D (2013) P-proteins in Arabidopsis are heteromeric structures involved in rapid sieve tube sealing. Front Plant Sci 4:51389. 10.3389/FPLS.2013.00225/BIBTEX 10.3389/fpls.2013.00225 PMC3700381 23840197

[CR34] Johnsson N, Varshavsky A (1994) Split ubiquitin as a sensor of protein interactions in vivo. Proc Natl Acad Sci U S A 91(22):10340–10344. 10.1073/pnas.91.22.10340 7937952 10.1073/pnas.91.22.10340 PMC45015

[CR35] Keller Y, Bouvier F, d’Harlingue A, Camara B (1998) Metabolic compartmentation of plastid prenyllipid biosynthesis. Evidence for the involvement of a multifunctional geranylgeranyl reductase. Eur J Biochem 251(1–2):413–417. 10.1046/j.1432-1327.1998.2510413.x 9492312 10.1046/j.1432-1327.1998.2510413.x

[CR36] Kim EY, Park KY, Seo YS, Kim WT (2016) Arabidopsis small rubber particle protein homolog SRPs Play dual roles as positive factors for tissue growth and development and in drought stress responses. Plant Physiol 170(4):2494–2510. 10.1104/pp.16.00165 26903535 10.1104/pp.16.00165 PMC4825120

[CR37] Kimura E, Abe T, Murata K, Kimura T, Otoki Y, Yoshida T, Miyazawa T, Nakagawa K (2018) Identification of OsGGR2, a second geranylgeranyl reductase involved in α-tocopherol synthesis in rice. Sci Rep 8(1):1870. 10.1038/s41598-018-19527-3 29382838 10.1038/s41598-018-19527-3 PMC5789843

[CR38] Kirschner J, Štěpánek J, Černý T, De Heer P, van Dijk PJ (2013) Available ex situ germplasm of the potential rubber crop Taraxacum koksaghyz belongs to a poor rubber producer, T. brevicorniculatum (Compositae–Crepidinae). Genet Resour Crop Evol 60(2):455–471. 10.1007/s10722-012-9848-0

[CR39] Konno K (2011) Plant latex and other exudates as plant defense systems: roles of various defense chemicals and proteins contained therein. Phytochemistry 72(13):1510–1530. 10.1016/J.PHYTOCHEM.2011.02.016 21450319 10.1016/j.phytochem.2011.02.016

[CR40] Laibach N, Hillebrand A, Twyman RM, Prüfer D, Schulze Gronover C (2015a) Identification of a *Taraxacum brevicorniculatum* rubber elongation factor protein that is localized on rubber particles and promotes rubber biosynthesis. Plant J 82(4):609–620. 10.1111/tpj.12836 25809497 10.1111/tpj.12836

[CR41] Laibach N, Post J, Twyman RM, Schulze Gronover C, Prüfer D (2015b) The characteristics and potential applications of structural lipid droplet proteins in plants. J Biotechnol 201:15–27. 10.1016/J.JBIOTEC.2014.08.020 25160916 10.1016/j.jbiotec.2014.08.020

[CR42] Laibach N, Schmidl S, Müller B, Bergmann M, Prüfer D, Schulze Gronover C (2018) Small rubber particle proteins from *Taraxacum brevicorniculatum* promote stress tolerance and influence the size and distribution of lipid droplets and artificial poly( *cis* -1,4-isoprene) bodies. Plant J 93(6):1045–1061. 10.1111/tpj.13829 29377321 10.1111/tpj.13829

[CR43] Lin T, Xu X, Ruan J, Liu S, Wu S, Shao X, Wang X, Gan L, Qin B, Yang Y, Cheng Z, Yang S, Zhang Z, Xiong G, Huang S, Yu H, Li J (2018) Genome analysis of Taraxacum kok-saghyz Rodin provides new insights into rubber biosynthesis. Natl Sci Rev 5(1):78–87. 10.1093/nsr/nwx101

[CR44] Lin T, Xu X, Du H, Fan X, Chen Q, Hai C, Zhou Z, Su X, Kou L, Gao Q, Deng L, Jiang J, You H, Ma Y, Cheng Z, Wang G, Liang C, Zhang G, Yu H, Li J (2022) Extensive sequence divergence between the reference genomes of Taraxacum kok-saghyz and Taraxacum mongolicum. Sci China Life Sci 65(3):515–528. 10.1007/S11427-021-2033-2 34939160 10.1007/s11427-021-2033-2

[CR45] Lippold F, vom Dorp K, Abraham M, Hölzl G, Wewer V, Yilmaz JL, Lager I, Montandon C, Besagni C, Kessler F, Stymne S, Dörmann P (2012) Fatty acid phytyl ester synthesis in chloroplasts of Arabidopsis. Plant Cell 24(5):2001–2014. 10.1105/tpc.112.095588 22623494 10.1105/tpc.112.095588 PMC3442583

[CR46] Liu H, Ouyang B, Zhang J, Wang T, Li H, Zhang Y, Yu C, Ye Z (2012) Differential modulation of photosynthesis, signaling, and transcriptional regulation between tolerant and sensitive tomato genotypes under cold stress. PLoS ONE 7(11):e50785. 10.1371/JOURNAL.PONE.0050785 23226384 10.1371/journal.pone.0050785 PMC3511270

[CR47] Liu H, Liu J, Zhao MM, Chen JS (2015) Overexpression of ShCHL P in tomato improves seedling growth and increases tolerance to salt, osmotic, and oxidative stresses. Plant Growth Regul 77(2):211–221. 10.1007/S10725-015-0054-X

[CR48] Liu X, Yi X, Yang YR, Huang QQ (2021) A rice geranylgeranyl reductase is essential for chloroplast development. J Integr Agric 20(10):2592–2600. 10.1016/S2095-3119(20)63324-X

[CR49] Liu S, Chen Y, Han D, Tian X, Ma D, Jie X, Zhang J (2024) Extraction process and characterization of Taraxacum kok-saghyz (TKS) latex. Heliyon. 10.1016/j.heliyon.2024.e25351 38379982 10.1016/j.heliyon.2024.e25351 PMC10877186

[CR50] Lundquist PK, Poliakov A, Bhuiyan NH, Zybailov B, Sun Q, van Wijk KJ (2012) The functional network of the Arabidopsis plastoglobule proteome based on quantitative proteomics and genome-wide coexpression analysis. Plant Physiol 158(3):1172–1192. 10.1104/PP.111.193144 22274653 10.1104/pp.111.193144 PMC3291262

[CR51] Madeira F, Madhusoodanan N, Lee J, Eusebi A, Niewielska A, Tivey ARN, Lopez R, Butcher S (2024) The EMBL-EBI job dispatcher sequence analysis tools framework in 2024. Nucleic Acids Res 52(W1):W521–W525. 10.1093/NAR/GKAE241 38597606 10.1093/nar/gkae241 PMC11223882

[CR52] Moir GFJ (1959) Ultracentrifugation and staining of hevea latex. Nature 184(4699):1626–1628. 10.1038/1841626a0

[CR53] Müller B, Noll GA, Ernst AM, Rüping B, Groscurth S, Twyman RM, Kawchuk LM, Prüfer D (2010) Recombinant artificial forisomes provide ample quantities of smart biomaterials for use in technical devices. Appl Microbiol Biotechnol 88(3):689–698. 10.1007/s00253-010-2771-4 20665019 10.1007/s00253-010-2771-4

[CR54] Niephaus E, Müller B, van Deenen N, Lassowskat I, Bonin M, Finkemeier I, Prüfer D, Schulze Gronover C (2019) Uncovering mechanisms of rubber biosynthesis in Taraxacum koksaghyz – role of cis-prenyltransferase-like 1 protein. Plant J 100(3):591–609. 10.1111/tpj.14471 31342578 10.1111/tpj.14471

[CR55] Olbińska E, Trela-Makowej A, Larysz W, Orzechowska A, Szymańska R (2023) The effect of α-tocopherol incorporated into different carriers on the oxidative stability of oil in water (O/W) emulsions. Colloids Surf B Biointerfaces 230:113536. 10.1016/j.colsurfb.2023.113536 37696162 10.1016/j.colsurfb.2023.113536

[CR56] Orlova I, Nagegowda DA, Kish CM, Gutensohn M, Maeda H, Varbanova M, Fridman E, Yamaguchi S, Hanada A, Kamiya Y, Krichevsky A, Citovsky V, Pichersky E, Dudareva N (2009) The small subunit of snapdragon geranyl diphosphate synthase modifies the chain length specificity of tobacco geranylgeranyl diphosphate synthase in planta. Plant Cell 21(12):4002–4017. 10.1105/TPC.109.071282 20028839 10.1105/tpc.109.071282 PMC2814502

[CR57] Park MR, Cho EA, Rehman S, Yun S (2010) Expression of a sesame geranylgeranyl reductase cDNA is induced by light but repressed by abscisic acid and ethylene. Pak J Bot 42(3):1815–1825

[CR58] Parmryd I, Andersson B, Dallner G (1999) Protein prenylation in spinach chloroplasts. Proc Natl Acad Sci U S A 96(18):10074–10079. 10.1073/PNAS.96.18.10074 10468564 10.1073/pnas.96.18.10074 PMC17844

[CR59] Paysan-Lafosse T, Blum M, Chuguransky S, Grego T, Pinto BL, Salazar GA, Bileschi ML, Bork P, Bridge A, Colwell L, Gough J, Haft DH, Letunić I, Marchler-Bauer A, Mi H, Natale DA, Orengo CA, Pandurangan AP, Rivoire C, Bateman A (2023) Interpro in 2022. Nucleic Acids Res 51(D1):D418–D427. 10.1093/NAR/GKAC993 36350672 10.1093/nar/gkac993 PMC9825450

[CR60] Post J, van Deenen N, Fricke J, Kowalski N, Wurbs D, Schaller H, Eisenreich W, Huber C, Twyman RM, Prüfer D, Schulze Gronover C (2012) Laticifer-specific cis-prenyltransferase silencing affects the rubber, triterpene, and inulin content of Taraxacum brevicorniculatum. Plant Physiol 158(3):1406–1417. 10.1104/pp.111.187880 22238421 10.1104/pp.111.187880 PMC3291264

[CR61] Pütter KM, van Deenen N, Müller B, Fuchs L, Vorwerk K, Unland K, Bröker JN, Scherer E, Huber C, Eisenreich W, Prüfer D, Schulze Gronover C (2019) The enzymes OSC1 and CYP716A263 produce a high variety of triterpenoids in the latex of Taraxacum koksaghyz. Sci Rep 9(1):1–13. 10.1038/s41598-019-42381-w 30976052 10.1038/s41598-019-42381-w PMC6459903

[CR62] Reichel C, Johnsson N (2005) The split-ubiquitin sensor: measuring interactions and conformational alterations of proteins in vivo. Methods Enzymol 399:757–776. 10.1016/S0076-6879(05)99050-2 16338394 10.1016/S0076-6879(05)99050-2

[CR63] Riva-Roveda L, Escale B, Giauffret C, Périlleux C (2016) Maize plants can enter a standby mode to cope with chilling stress. BMC Plant Biol 16(1):212. 10.1186/s12870-016-0909-y 27716066 10.1186/s12870-016-0909-y PMC5050578

[CR64] Ruiz-Sola MÁ, Barja MV, Manzano D, Llorente B, Schipper B, Beekwilder J, Rodriguez-Concepcion M (2016a) A single Arabidopsis gene encodes two differentially targeted geranylgeranyl diphosphate synthase isoforms. Plant Physiol 172(3):1393–1402. 10.1104/pp.16.01392 27707890 10.1104/pp.16.01392 PMC5100792

[CR65] Ruiz-Sola MÁ, Coman D, Beck G, Barja MV, Colinas M, Graf A, Welsch R, Rütimann P, Bühlmann P, Bigler L, Gruissem W, Rodríguez-Concepción M, Vranová E (2016b) Arabidopsis GERANYLGERANYL DIPHOSPHATE SYNTHASE 11 is a hub isozyme required for the production of most photosynthesis-related isoprenoids. New Phytol 209(1):252–264. 10.1111/nph.13580 26224411 10.1111/nph.13580

[CR66] Salomé Abarca LF, Klinkhamer PGL, Choi YH (2019) Plant latex, from ecological interests to bioactive chemical resources. Planta Med 85(11–12):856–868. 10.1055/a-0923-8215 31137048 10.1055/a-0923-8215

[CR67] Schmidt FJ, Zimmermann MM, Wiedmann DR, Lichtenauer S, Grundmann L, Muth J, Twyman RM, Prüfer D, Noll GA (2020) The major floral promoter NtFT5 in tobacco (Nicotiana tabacum) is a promising target for crop improvement. Front Plant Sci. 10.3389/FPLS.2019.01666/FULL 31998348 10.3389/fpls.2019.01666 PMC6966700

[CR68] Seo SG, Kim JS, Yang YS, Jun BK, Kang SW, Lee GP, Kim W, Kim JB, Lee HU, Kim SH (2010) Cloning and characterization of the new multiple stress responsible gene i (MuSI) from sweet potato. Genes Genom 32(6):544–552. 10.1007/s13258-010-0093-7

[CR69] Shpilyov AV, Zinchenko VV, Shestakov SV, Grimm B, Lokstein H (2005) Inactivation of the geranylgeranyl reductase (ChlP) gene in the cyanobacterium Synechocystis sp. PCC 6803. Biochim Biophys Acta 1706(3):195–203. 10.1016/j.bbabio.2004.11.001 15694347 10.1016/j.bbabio.2004.11.001

[CR70] Soll J, Schultz G (1981) Phytol synthesis from geranylgeraniol in spinach chloroplasts. Biochem Biophys Res Commun 99(3):907–912. 10.1016/0006-291X(81)91249-3 7247947 10.1016/0006-291x(81)91249-3

[CR71] Stahl AM, Lüdtke LF, Grimaldi R, Gigante LM, Ribeiro APB (2024) Characterization and stability of α-tocopherol loaded solid lipid nanoparticles formulated with different fully hydrogenated vegetable oils. Food Chem 1(439):138149. 10.1016/j.foodchem.2023.138149 10.1016/j.foodchem.2023.138149 38064825

[CR72] Stolze A, Wanke A, van Deenen N, Geyer R, Prüfer D, Schulze Gronover C (2017) Development of rubber-enriched dandelion varieties by metabolic engineering of the inulin pathway. Plant Biotechnol J 15(6):740–753. 10.1111/pbi.12672 27885764 10.1111/pbi.12672 PMC5425391

[CR73] Suire C, Bouvier F, Backhaus RA, Bégu D, Bonneu M, Camara B (2000) Cellular localization of isoprenoid biosynthetic enzymes in Marchantia polymorpha. Uncovering a new role of oil bodies. Plant Physiol 124(3):971–978. 10.1104/PP.124.3.971 11080275 10.1104/pp.124.3.971 PMC59197

[CR74] Takahashi K, Takabayashi A, Tanaka A, Tanaka R (2014) Functional analysis of light-harvesting-like protein 3 (LIL3) and its light-harvesting chlorophyll-binding motif in Arabidopsis. J Biol Chem 289(2):987–999. 10.1074/jbc.M113.525428 24275650 10.1074/jbc.M113.525428 PMC3887221

[CR75] Takaya A, Zhang Y-W, Asawatreratanakul K, Wititsuwannakul D, Wititsuwannakul R, Takahashi S, Koyama T (2003) Cloning, expression and characterization of a functional cDNA clone encoding geranylgeranyl diphosphate synthase of Hevea brasiliensis. Biochimica Et Biophysica Acta (BBA) Gene Struct Expr 1625(2):214–220. 10.1016/S0167-4781(02)00602-4 10.1016/s0167-4781(02)00602-4 12531482

[CR76] Tamura K, Stecher G, Kumar S (2021) MEGA11: molecular evolutionary genetics analysis version 11. Mol Biol Evol 38(7):3022–3027. 10.1093/MOLBEV/MSAB120 33892491 10.1093/molbev/msab120 PMC8233496

[CR77] Tanaka R, Oster U, Kruse E, Rüdiger W, Grimm B (1999) Reduced activity of geranylgeranyl reductase leads to loss of chlorophyll and tocopherol and to partially geranylgeranylated chlorophyll in transgenic tobacco plants expressing antisense RNA for geranylgeranyl reductase. Plant Physiol 120(3):695. 10.1104/PP.120.3.695 10398704 10.1104/pp.120.3.695 PMC59307

[CR78] Tanaka R, Rothbart M, Oka S, Takabayashi A, Takahashi K, Shibata M, Myouga F, Motohashi R, Shinozaki K, Grimm B, Tanaka A (2010) LIL3, a light-harvesting-like protein, plays an essential role in chlorophyll and tocopherol biosynthesis. Proc Natl Acad Sci USA 107(38):16721–16725. 10.1073/pnas.1004699107 20823244 10.1073/pnas.1004699107 PMC2944722

[CR79] Tangpakdee J, Tanaka Y, Ogura K, Koyama T, Wititsuwannakul R, Wititsuwannakul D, Asawatreratanakul K (1997) Isopentenyl diphosphate isomerase and prenyl transferase activities in bottom fraction and c-serum from Hevea latex. Phytochemistry 45(2):261–267. 10.1016/S0031-9422(96)00837-0

[CR80] Tholl D (2015) Biosynthesis and biological functions of terpenoids in plants. Adv Biochem Eng Biotechnol 148:63–106. 10.1007/10_2014_295/COVER 25583224 10.1007/10_2014_295

[CR81] Turnbull D, Hemsley PA (2017) Fats and function: protein lipid modifications in plant cell signalling. Curr Opin Plant Biol 40:63–70. 10.1016/J.PBI.2017.07.007 28772175 10.1016/j.pbi.2017.07.007

[CR82] Unland K, Pütter KM, Vorwerk K, van Deenen N, Twyman RM, Prüfer D, Schulze Gronover C (2018) Functional characterization of squalene synthase and squalene epoxidase in Taraxacum koksaghyz. Plant Direct 2(6):1–15. 10.1002/pld3.63 10.1002/pld3.63 PMC6508512 31245726

[CR83] Valentin HE, Lincoln K, Moshiri F, Jensen PK, Qi Q, Venkatesh TV, Karunanandaa B, Baszis SR, Norris SR, Savidge B, Gruys KJ, Last RL (2006) The Arabidopsis vitamin E pathway gene5-1 mutant reveals a critical role for phytol kinase in seed tocopherol biosynthesis. Plant Cell 18(1):212–224. 10.1105/tpc.105.037077 16361393 10.1105/tpc.105.037077 PMC1323494

[CR84] van Wijk KJ, Kessler F (2017) Plastoglobuli: plastid microcompartments with integrated functions in metabolism, plastid developmental transitions, and environmental adaptation. Annu Rev Plant Biol 68(1):253–289. 10.1146/annurev-arplant-043015-111737 28125283 10.1146/annurev-arplant-043015-111737

[CR85] Vidi P-A, Kanwischer M, Baginsky S, Austin JR, Csucs G, Dörmann P, Kessler F, Bréhélin C (2006) Tocopherol cyclase (VTE1) localization and vitamin E accumulation in chloroplast plastoglobule lipoprotein particles. J Biol Chem 281(16):11225–11234. 10.1074/jbc.M511939200 16414959 10.1074/jbc.M511939200

[CR86] vom Dorp K, Hölzl G, Plohmann C, Eisenhut M, Abraham M, Weber APM, Hanson AD, Dörmann P (2015) Remobilization of phytol from chlorophyll degradation is essential for tocopherol synthesis and growth of Arabidopsis. Plant Cell 27(10):2846–2859. 10.1105/tpc.15.00395 26452599 10.1105/tpc.15.00395 PMC4682318

[CR87] Wahler D, Schulze Gronover C, Richter C, Foucu F, Twyman RM, Moerschbacher BM, Fischer R, Muth J, Prüfer D (2009) Polyphenoloxidase silencing affects latex coagulation in Taraxacum species. Plant Physiol 151(1):334–346. 10.1104/pp.109.138743 19605551 10.1104/pp.109.138743 PMC2736003

[CR88] Wahler D, Colby T, Kowalski NA, Harzen A, Wotzka SY, Hillebrand A, Fischer R, Helsper J, Urgen Schmidt J, Schulze Gronover C, Prüfer D (2012) Proteomic analysis of latex from the rubber-producing plant Taraxacum brevicorniculatum. Proteomics 12:901–905. 10.1002/pmic.201000778 22539439 10.1002/pmic.201000778

[CR89] Wang G, Dixon RA (2009) Heterodimeric geranyl(geranyl)diphosphate synthase from hop (Humulus lupulus) and the evolution of monoterpene biosynthesis. Proc Natl Acad Sci U S A 106(24):9914–9919. 10.1073/pnas.0904069106 19482937 10.1073/pnas.0904069106 PMC2701037

[CR90] Wang P, Li C, Wang Y, Huang R, Sun C, Xu Z, Zhu J, Gao X, Deng X, Wang P (2014) Identification of a geranylgeranyl reductase gene for chlorophyll synthesis in rice. Springerplus 3(1):201. 10.1186/2193-1801-3-201 24809003 10.1186/2193-1801-3-201 PMC4008729

[CR91] Wang D, Xie Q, Sun Y, Tong Z, Chang L, Yu L, Zhang X, Yuan B, He P, Jin X, Dong Y, Li H, Montoro P, Wang X (2019) Proteomic landscape has revealed small rubber particles are crucial rubber biosynthetic machines for ethylene-stimulation in natural rubber production. Int J Mol Sci 2019(20):5082. 10.3390/IJMS20205082 10.3390/ijms20205082 PMC6829444 31614967

[CR92] Wang L, He H, Wang J, Meng Z, Wang L, Jin X, Zhang J, Du P, Zhang L, Wang F, Li H, Xie Q (2024) Genome-wide identification of the geranylgeranyl pyrophosphate synthase (GGPS) gene family associated with natural rubber synthesis in Taraxacum kok-saghyz L. Rodin. Plants 13(19):2788. 10.3390/PLANTS13192788 39409658 10.3390/plants13192788 PMC11478434

[CR93] Westfall PJ, Pitera DJ, Lenihan JR, Eng D, Woolard FX, Regentin R, Horning T, Tsuruta H, Melis DJ, Owens A, Fickes S, Diola D, Benjamin KR, Keasling JD, Leavell MD, McPhee DJ, Renninger NS, Newman JD, Paddon CJ (2012) Production of amorphadiene in yeast, and its conversion to dihydroartemisinic acid, precursor to the antimalarial agent artemisinin. Proc Natl Acad Sci U S A 109(3):E111-118. 10.1073/pnas.1110740109 22247290 10.1073/pnas.1110740109 PMC3271868

[CR94] Wolters SM, Benninghaus VA, Roelfs K-U, van Deenen N, Twyman RM, Prüfer D, Schulze Gronover C (2023) Overexpression of a pseudo-etiolated-in-light-like protein in Taraxacum koksaghyz leads to a pale green phenotype and enables transcriptome-based network analysis of photomorphogenesis and isoprenoid biosynthesis. Front Plant Sci 14:1228961. 10.3389/FPLS.2023.1228961/BIBTEX 37841614 10.3389/fpls.2023.1228961 PMC10569127

[CR95] Wolters SM, Laibach N, Riekötter J, Roelfs K-U, Müller B, Eirich J, Twyman RM, Finkemeier I, Prüfer D, Schulze Gronover C (2024) The interaction networks of small rubber particle proteins in the latex of Taraxacum koksaghyz reveal diverse functions in stress responses and secondary metabolism. Front Plant Sci 15:1498737. 10.3389/FPLS.2024.1498737 39735776 10.3389/fpls.2024.1498737 PMC11671276

[CR96] Wu Y, Dong G, Luo F, Xie H, Li X, Yan J (2024) TkJAZs-TkMYC2-TkSRPP / REF regulates the biosynthesis of natural rubber in Taraxacum kok-saghyz. Plants (Basel) 13(15):2034. 10.3390/plants13152034 39124151 10.3390/plants13152034 PMC11314035

[CR97] Xu T, Li Yi, Liu X, Yang X, Huang Z, Xing J, Liang C, Li J, Tan Y, Zhang S, Qi J, Ye D, li Z, Cao J, Tang C, Liu K (2025) Rubber biosynthesis drives the biogenesis and development of rubber particles, the rubber-producing organelles. Plant Biotechnol J. 10.1111/pbi.70052 40112038 10.1111/pbi.70052 PMC12120880

[CR98] Zhou F, Pichersky E (2020) The complete functional characterisation of the terpene synthase family in tomato. New Phytol 226(5):1341–1360. 10.1111/NPH.16431 31943222 10.1111/nph.16431 PMC7422722

[CR99] Zhou Y, Gong Z, Yang Z, Yuan Y, Zhu J, Wang M, Yuan F, Wu S, Wang Z, Yi C, Xu T, Ryom M, Gu M, Liang G (2013) Mutation of the light-induced yellow leaf 1 gene, which encodes a geranylgeranyl reductase, affects chlorophyll biosynthesis and light sensitivity in rice. PLoS ONE. 10.1371/journal.pone.0075299 24058671 10.1371/journal.pone.0075299 PMC3769248

[CR100] Zhou F, Wang C-Y, Gutensohn M, Jiang L, Zhang P, Zhang D, Dudareva N, Lu S (2017) A recruiting protein of geranylgeranyl diphosphate synthase controls metabolic flux toward chlorophyll biosynthesis in rice. Proc Natl Acad Sci U S A 114(26):6866–6871. 10.1073/pnas.1705689114 28607067 10.1073/pnas.1705689114 PMC5495272

