# Supplementary material for: TkSRPP3/4 interactors TkGGR1 and TkLIL3 link plastid-like organelles with isoprenoid metabolism in Taraxacum koksaghyz latex
Source: Plant Cell Rep. 2025 Jun 24;44(7):155. doi: 10.1007/s00299-025-03537-3 (PMC12187845; doi:10.1007/s00299-025-03537-3)
Supplement: Supplementary file 1 — Supplementary file1 (DOCX 6140 KB) [file 299_2025_3537_MOESM1_ESM.docx]

Supplementary Material

**Table 1: List of primers used in this study.**

| **Target** | **Primer name** | **Sequence [5’→ 3’]** | **Primer efficiency for qPCR** |
| --- | --- | --- | --- |
| *GGR1* | TkGGR1-NcoI-fwd | AAACCATGGCCGTCGTCATTCAC |  |
|  | TkGGR1-NotI-rev | AAAGCGGCCGCTTAAACGAACTTCGCAAG |  |
|  | TkGGR1 XhoI fwd | AAACTCGAGCAACAATGGCCGTCGTCATTCAC |  |
|  | TkGGR1 XbaI rev | AAATCTAGATTAAACGAACTTCGCAAGAAC |  |
|  | TkGGR1ns-NotI-rev | AAAGCGGCCGCACGAACTTCGCAAG |  |
|  | TkGGR1-RNAi-NcoI | ATACCATGGCCGCTTCTTCTACTTCAAAC |  |
|  | TkGGR1-RNAi-XhoI | ATACTCGAGCCGGTGGGGCTGCGCTCA |  |
|  | TkGGR1nS XbaI rv | AAAtctagaAACGAACTTCGCAAGAACAC |  |
|  | GGR1 qPCR fw | GGAGACGCCGCCGGGTATG | 103.9% |
|  | GGR1 qPCR rv | ACCCTCCTTCCGACGCTTTCAC |  |
| *TkGGPS6* | TkGGPS6-NcoI-fwd | AAACCATGGAGAGATCTCTGAATTTG |  |
|  | TkGGPS6-NotI-rev | AAAGCGGCCGCTTAGTTCTGCCGGTAAGC |  |
|  | TkGGPS6ns-NotI-rev | AAAGCGGCCGCGAGTTCTGCCGGTAAGC |  |
|  | TkGGPS6 qPCR fw | GGGAAGACCGCCGGGAAAGAC | 104.8% |
|  | TkGGPS6 qPCR rv | CGCCAACAACTCCTCCGCAAAC |  |
| *GGR2* | TkGGR2-NcoI-fwd | AAACCATGGCCTCCGTCGCTC |  |
|  | TkGGR2-XhoI-rev | AAACTCGAGTTAAATCTTGGCCATCTCC |  |
|  | GGR2 qPCR fw | GCTCCGTACGTCCTCCAT | 92.8% |
|  | GGR2 qPCR rv | CGTCGGCTCCGATTACTG |  |
|  | GGR2 RNAi NcoI fw | CCATGGCCTCCGTCGCTCTC |  |
|  | GGR2 RNAi XhoI rv | CTCGAGGGGCTGGACTTACCGG |  |
| *TkLIL3* | TkLIL3-NcoI-fwd | AAACCATGGCTATGTCTTCATCAATGGCTTTGTTC |  |
|  | TkLIL3-NotI-rev | AAAGCGGCCGCTCATTCTTTTTTCTCATCTTGCCAA |  |
|  | TkLIL3ns-NotI-rev | AAAGCGGCCGCCATTCTTTTTTCTCATCTTGCCAA |  |
|  | TkLIL3 qPCR fw | CCCTGTAGTTTTCGACACTTCC | 94.7% |
|  | TkLIL3 qPCR rv | GTAAGCCATGAAGAACCCCAC |  |
| *TkRP* | TkRP qPCR fw | cgtcgatctcaaggatgttgtc | 95.7% |
|  | TkRP qPCR rv | ggagctttgagaagaaccaacg |  |
| *TkEF1 α* | TkEF1α qPCR fw | cgagagattcgagaaggaagc | 104.8% |
|  | TkEF1α qPCR rv | ctgtgcagtagtacttggtgg |  |
|  | mVenus XbaI fw | AAAtctagaACAATGGTGAGCAAGGGCG |  |
|  | mVenus XmaI rv | AAACCCGGGTTACTTGTACAGCTCGTCCATGC |  |
|  | TP Rubisco NcoI fw | AAACCatggcttcctcagttctttc |  |
|  | TP Rubisco XhoI rev | aaactcgagctcaaatcaggcaggtatg |  |
|  | 35S Prom fw | ATCTCCACTGACGTAAGG |  |
|  | 35S Term rv | TGCTCAACACATGAGCGAAACC |  |
|  | Cerulean seq rv | GTATCTTGCAAAGCATTGTACTC |  |
|  | eYFPrevscreen | agcttgccgtaggtggc |  |
|  | mEmerald screen rv | AAGTAGTGACAAGTGTTGG |  |
|  | PMet17 seq fw | GTCTTTTCATCTACTATTTCC |  |
|  | Cub seq rv | GTAATCAGACAGCGTTCTACC |  |
|  | Nua/Nui Seq fw | CAAGACTTTGACCGGTAAAACC |  |
|  | pAGD fw | ctagaactagtggatcccccatc |  |

**Table 2: Overview of TkGGR sequences identified in this study.** Corresponding IDs from the two published T. koksaghyz genomes are listed. The final column indicates whether a chloroplast transit peptide (TP) was predicted for the deduced protein sequences using TargetP2.0.

| **Name** | **Gene ID Lin *et al.*, 2018** | **Gene ID Lin *et al.*, 2022** | **GWHT ID *Lin et al.*, 2022** | **Chloroplast TP predicted** |
| --- | --- | --- | --- | --- |
| GGR1a | evm.model.utg13566.2 | GWHBCHF00000004: 106367953-106369331 | GWHTBCHF019673 | Yes |
| GGR1b | evm.model.utg13086.2; evm.model.utg11343.5 | GWHBCHF00000006: 99634824-99636201 | - | Yes |
| GGR2 | evm.model.utg4815.2 | - | - | Yes |
| GGR3 | - | GWHBCHF00000004: 85983351- 85984316 | GWHTBCHF018730 | No |
| GGR4 | evm.model.utg27960.3 | - | - | No |
| GGR5 | evm.model.utg10254.5 | - | - | Yes |
| GGR6 | evm.model.utg27612.2 | - | - | No |
| GGR7 | evm.model.utg27612.3 | - | - | Yes |


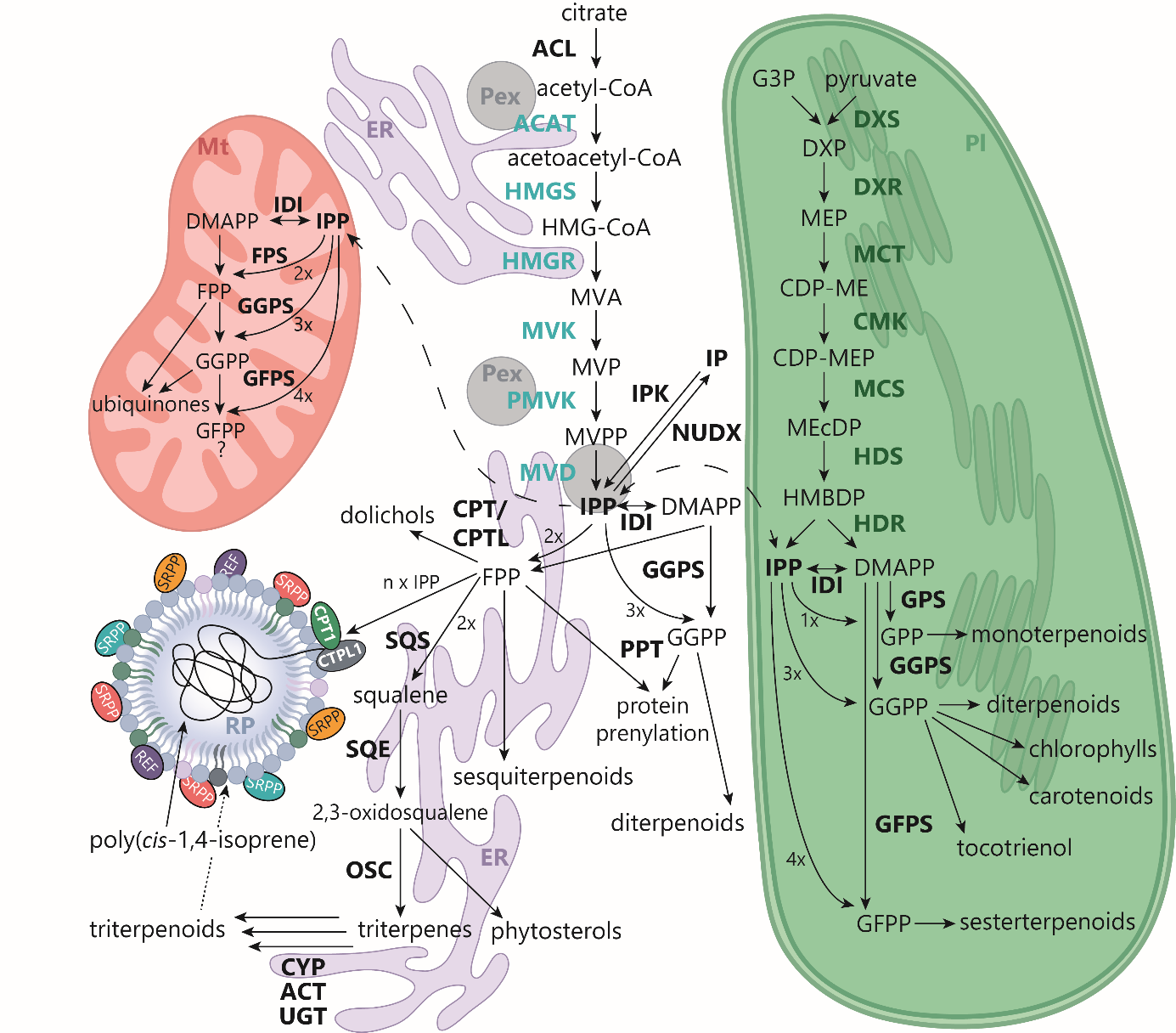


**Figure 9**: **Illustration of the isoprenoid metabolic network in plant cells.** Enzymes are written in bold. Enzymes written in green represent those belonging to the plastidial methylerythritol (MEP) pathway, enzymes written in blue belong to the cytoplasmic mevalonate (MVA) pathway. Dashed arrows indicate an unclear mechanism of metabolite transport. The illustration of poly(cis-1,4-isoprene) synthesis refers to the current model for T. koksaghyz. Dotted arrow indicates an unclear connection between triterpenoid synthesis and their detection inside rubber particles.ACAT: acetyl-CoA C-acetyltransferase, ACL: ATP-citrate lyase/synthase, ACT: acyltransferase, CMK: 4- diphosphocytidyl-2-C-methyl-D-erythritol kinase, CPT: cis-prenyltransferase, CPTL: cis-prenyltransferase-like, CYP: cytochrome P450 monooxygenase, DXR: 1-deoxy-D-xylulose-5-phosphate reductoisomerase, DXS: 1-deoxy- D-xylulose-5-phosphate synthase, ER: endoplasmic reticulum, FPS: farnesyl diphosphate synthase, GFPS:geranylfarnesyl diphosphate synthase, GGPS: geranylgeranyl diphosphate synthase, G3P: glyceraldehyde 3- phosphate, GPS: geranyl diphosphate synthase, HDR: 4-hydroxy-3-methylbut-2-en-1-yl diphosphate reductase, HDS: (E)-4-hydroxy-3-methylbut-2-enyl-diphosphate synthase, HMGR: hydroxymethylglutaryl-CoA reductase, HMGS: hydroxymethylglutaryl-CoA synthase, IDI: isopentenyl diphosphate (IPP) δ-isomerase, IPK: isopentenyl phosphate kinase, MCS: 2-C-methyl-D-erythritol 2,4-cyclodiphosphate synthase, MCT: 2-C-methyl-D-erythritol 4- phosphate cytidylyltransferase, Mt: mitochondrion, MVD: diphosphomevalonate decarboxylase, MVK: mevalonate kinase, NUDX: Nudix hydrolase, OSC: oxidosqualene cyclase, Pex: peroxisome, Pl: plastid, PMVK:phosphomevalonate kinase, PPT: protein prenyltransferase, REF: rubber elongation factor, RP: rubber particle, SRPP: small rubber particle protein, SQE: squalene epoxidase, SQS: squalene synthase, UGT: UDP-glucosyltransferase. Abbreviations of metabolites: DMAPP: dimethylallyl diphosphate, DXP: 1-deoxy-D-xylulose-5-phosphate, DXR : 1-deoxy-D-xylulose-5-phosphate reductoisomerase, DXS: 1-deoxy-d-xylulose-5-phosphate synthase, FPP: farnesyl diphosphate, GFPP: geranylfarnesyl diphosphate, GGPP: geranylgeranyl diphosphate, GPP: geranyl diphosphate, HMBDP: (E)-4-hydroxy-3-methylbut-2-enyl-diphosphate, HMG-CoA: 3-hydroxy-3-methylglutaryl-CoA, IPP: isopentenyl diphosphate, MEcDP: 2-C-methyl-D-erythritol 2,4-cyclodiphosphate, MEP: methylerythritol, MVA: mevalonate. Modified from the PhD thesis of Silvia Melissa Wolters, Münster 2025.


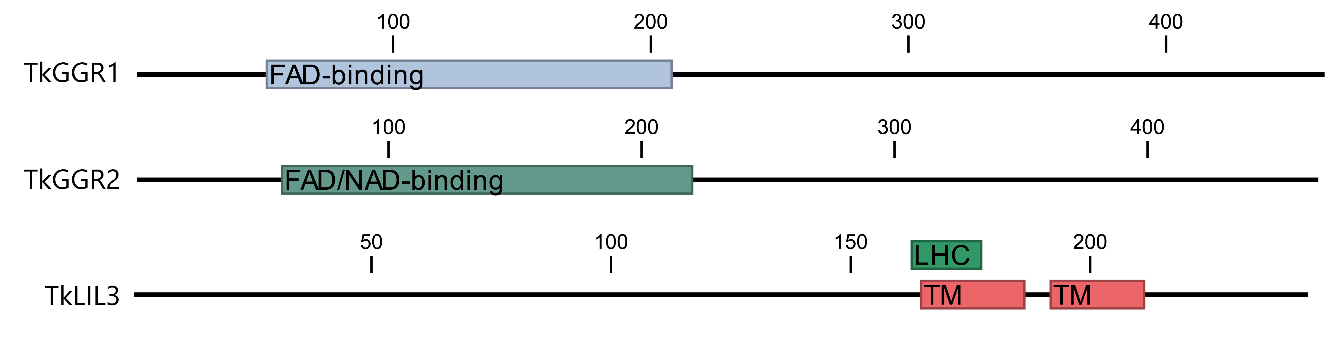


**Figure 10: TkGGR1, TkGGR2 and TkLIL3 contain typical protein domains**. Schematic overview of TkGGR1, TkGGR2 and TkLIL3 proteins showing domains predicted using InterPro. Both GGR proteins were predicted to contain a FAD or FAD/NAD-binding domain necessary for the catalysis of redox reactions. TkLIL3 features two transmembrane domains, the first containing the eponymous LHC motif. TM, transmembrane domain; LHC, light-harvesting complex motif.


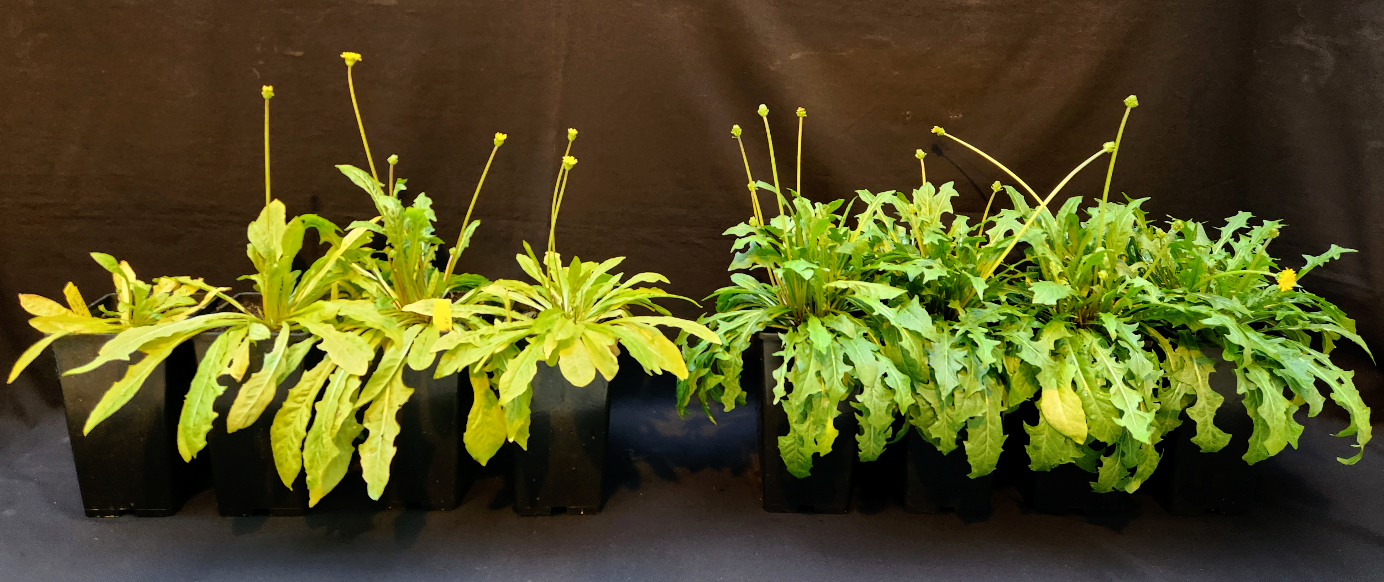


**Figure 11**: **Three lines of TbGGR2-RNAi lines with either pale green (high suppression) or dark green leaves (moderate suppression**). The plants were grown under controlled greenhouse conditions for 14 weeks.

*
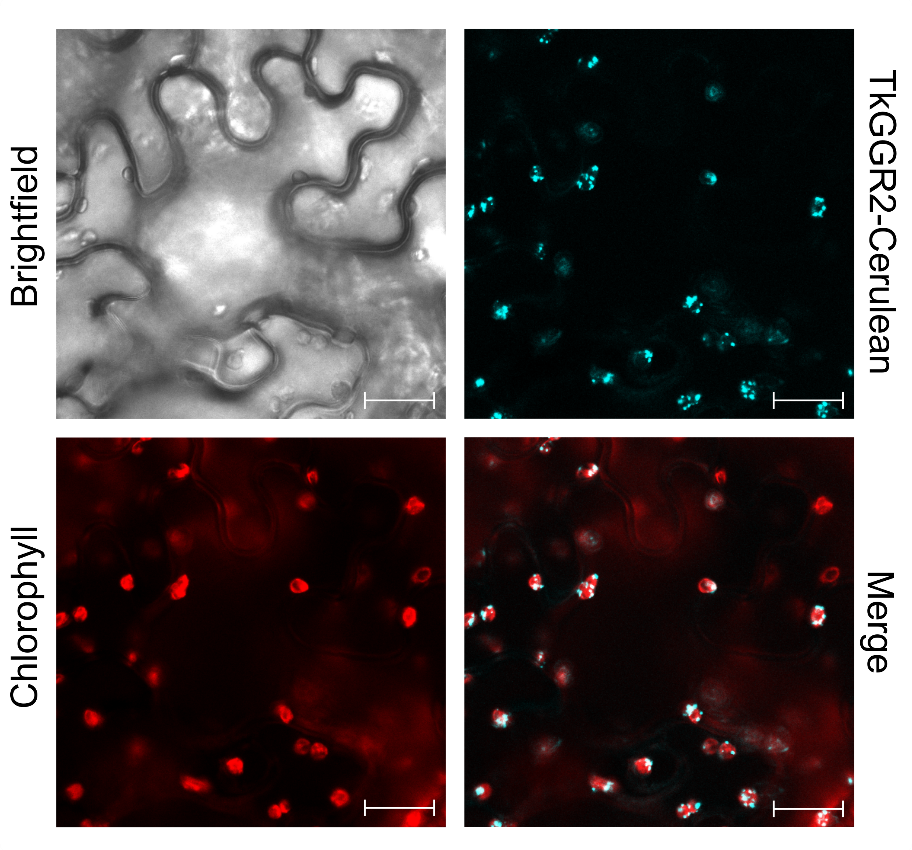
*

**Figure 12**: **TkGGR2 is found in punctuate structures within leaf chloroplasts when expressed in *N. benthamiana* epidermal cells.** Punctuate TkGGR2–Cerulean fluorescence (cyan) within chloroplasts marked by chlorophyll autofluorescence in *N. benthamiana*. Microscopic images of *N. benthamiana* leaf epidermal cells transiently expressing a TkGGR2–Cerulean fusion protein, with chlorophyll autofluorescence shown in red. Scale bars = 20 µm.


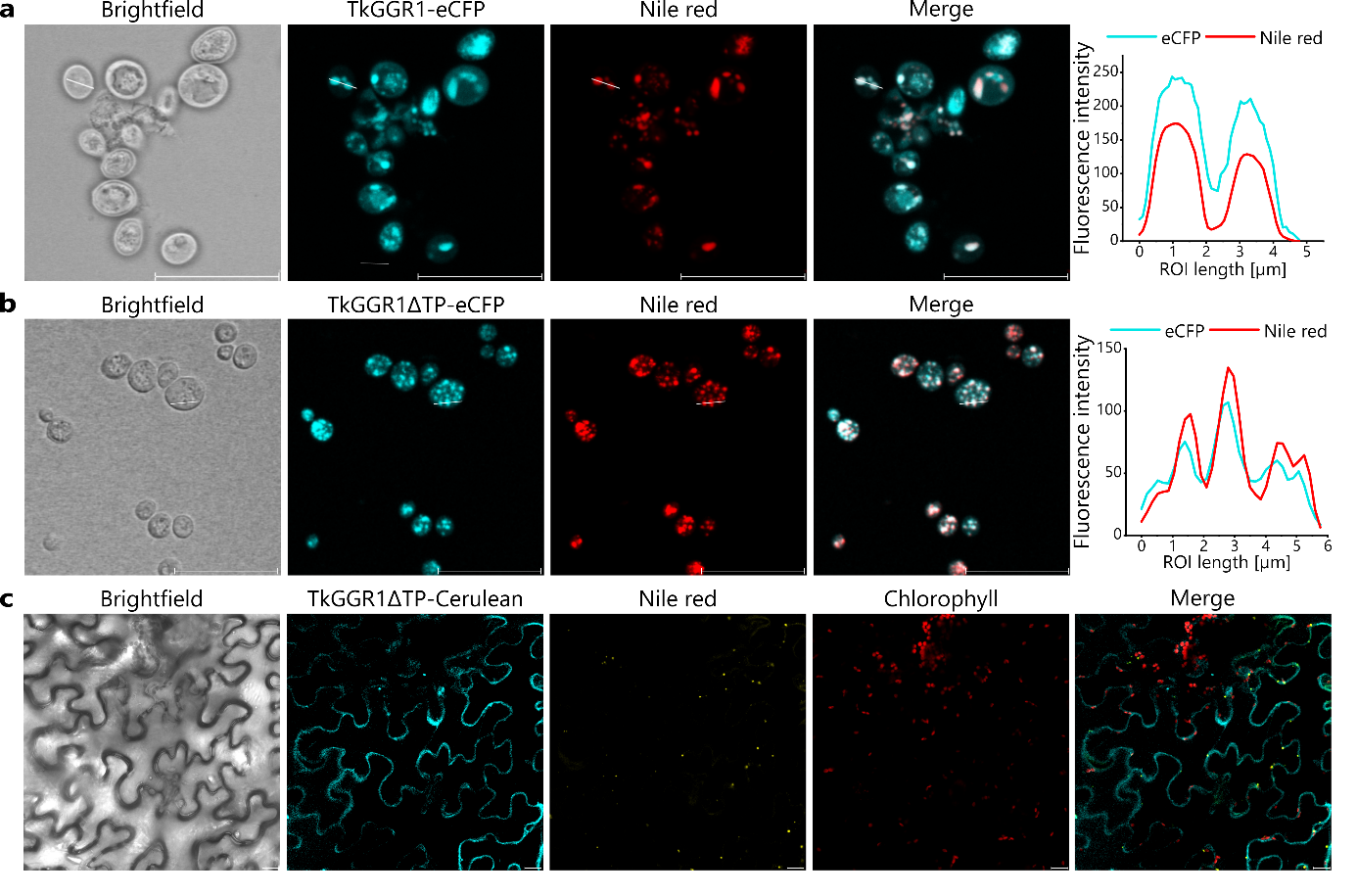


**Figure 13: TkGGR1 associates with lipid droplets in S. cerevisiae, but not with cytoplasmic lipid droplets in N. benthamiana.** a, b. S. cerevisiae cells expressing TkGGR1 (a) or TkGGR1 missing its chloroplast transit peptide (TP) (TkGGR1∆TP) (b) C-terminally fused to enhanced cyan fluorescent protein (eCFP) (cyan). Lipid droplets (LDs) were stained with Nile red (red). Fluorescence intensities in regions of interest are shown on the right and indicate overlapping TkGGR1(∆TP)-eCFP and Nile red signals. c. TkGGR1∆TP–Cerulean is not imported into chloroplasts and does not associate with cytoplasmic LDs in N. benthamiana. N. benthamiana leaf epidermal cells expressing a TkGGR1∆TP C-terminal Cerulean fusion (cyan) and AtLEC2 to induce LD formation. LDs were stained with Nile red (yellow). Chlorophyll autofluorescence is shown in red. Scale bars = 20 µm.


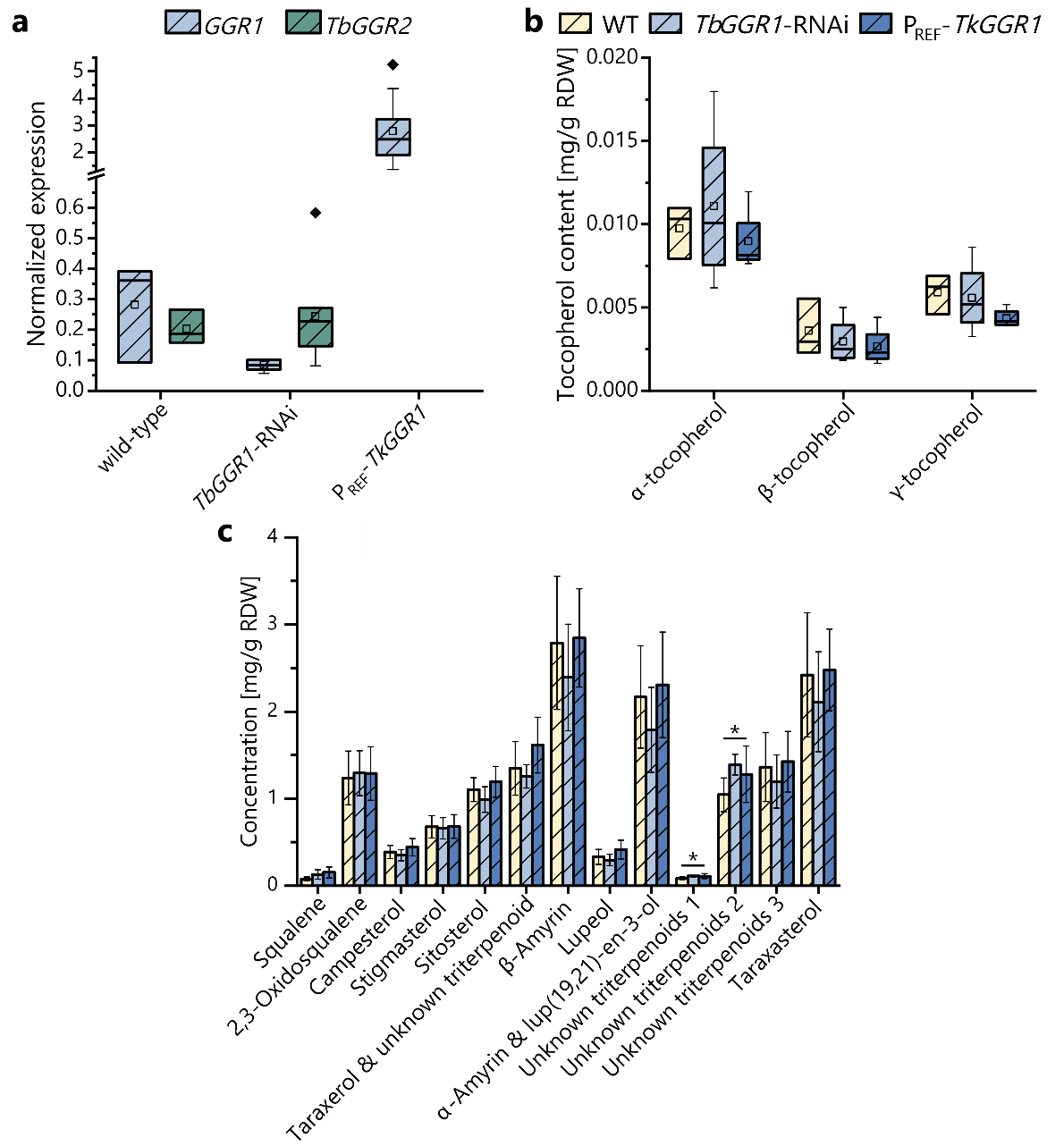


**Figure 14: *T. brevicorniculatum* P_REF_-*TkGGR1* and *TbGGR1*-RNAi lines do not differ from the wild-type in terms of root tocopherol content, but P_REF_-*TkGGR1* roots produce higher levels of two triterpenoids.** a. *GGR1* expression is reduced in the latex of *T. brevicorniculatum TbGGR1*-RNAi plants, whereas *TbGGR2* expression remains unaffected. *GGR1* expression levels are much higher in P_REF_-*TkGGR1* lines than in wild-type controls. Normalized gene expression levels in latex of 14-week-old *T. brevicorniculatum* plants. In P_REF_-*TkGGR1* lines, only *GGR1* expression was examined. Expression levels were normalized against *elongation factor-1 α* (*TbEF1α*) and *ribosomal protein L27* (*TbRP*). b. Root tocopherol levels do not differ between 14-week-old *TbGGR1*-RNAi, P_REF_-*TkGGR1* lines and wild-type *T. brevicorniculatum* controls. Statistical significance was determined using two-sample *t*-tests (*p < 0.05). c. Two unknown triterpenoids are more abundant in the roots of *T. brevicorniculatum* P_REF_-*TkGGR1* lines compared to wild-type controls. Quantification of triterpenoids and phytosterols in *T. brevicorniculatum* wild-type, *TbGGR1*-RNAi and P_REF_-*TkGGR1* roots. Data are means ± standard deviations. Statistical significance was determined using two-sample *t*-tests, or the Wilcoxon signed ranks test when data were not normally distributed (*p < 0.05). Multiple individual plants from different lines were analyzed: wild-type n=3, *TbGGR1*-RNAi n=8, P_REF_-*TkGGR1* n=10.


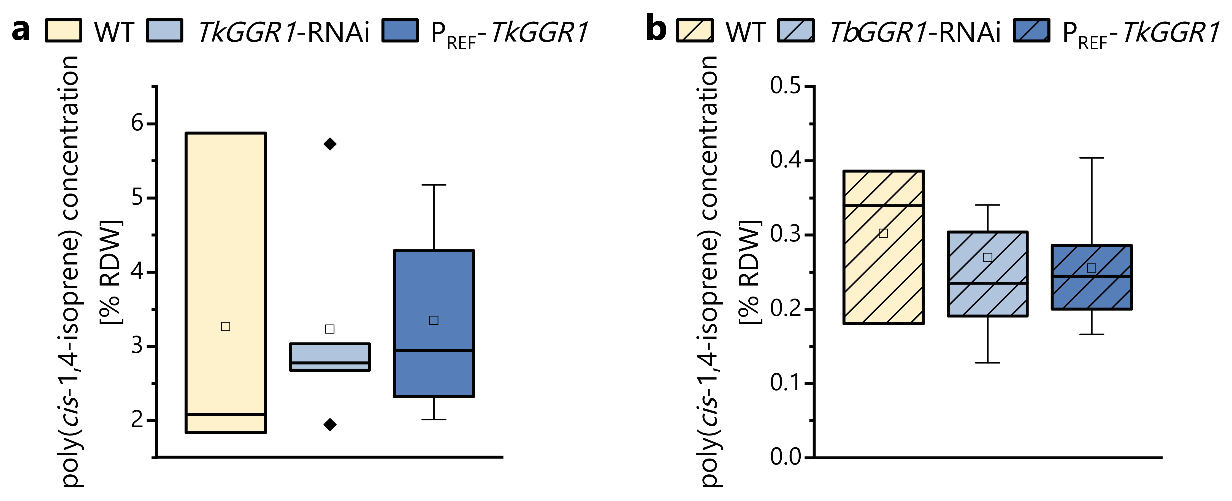


**Figure 15: Natural rubber content is unaffected in T. koksaghyz and T. brevicorniculatum plants with modified GGR1 expression compared to wild-type controls.** Quantification of poly(cis-1,4-isoprene) in roots of 14-week-old (a) T. koksaghyz and (b) T. brevicorniculatum P_REF_-TkGGR1 and GGR1-RNAi lines and wild-type plants. The concentration is shown as the percentage of root dry weight (RDW). Boxplots represent values from multiple individual plants: Tk wild-type n=3, TkGGR1-RNAi n=5, Tk P_REF_-TkGGR1 n=5, Tb wild-type n=3, TbGGR1-RNAi n=8, Tb P_REF_-TkGGR1 n=10. Statistical significance was determined using two-sample t-tests, or the Wilcoxon signed ranks test when data were not normally distributed.


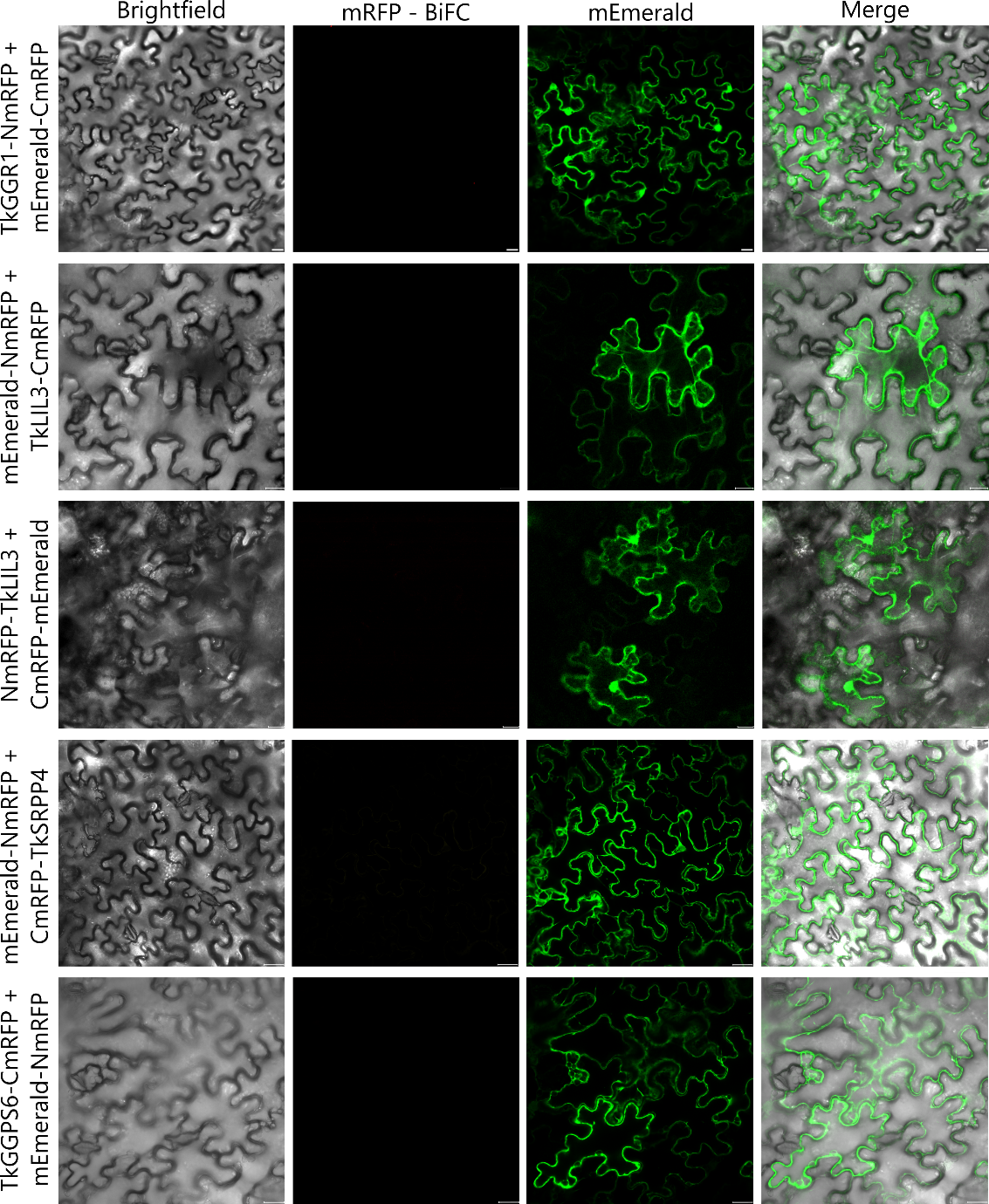


**Figure 16: Proteins investigated in this study do not interact nonspecifically with mEmerald in bimolecular fluorescence complementation assays.** Test proteins N- or C-terminally fused to the N- or C-terminal part of monomeric red fluorescent protein (NmRFP or CmRFP) were transiently co-expressed in N. benthamiana with mEmerald N- or C-terminally fused to the other part of mRFP. These combinations served as negative controls. Missing mRFP fluorescence indicates the absence of protein interaction and mEmerald fluorescence indicates successful gene expression. Scale bars = 20 µm.


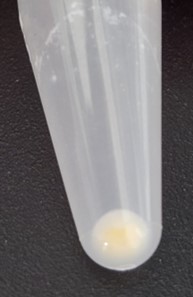


**Figure 17: The pellet phase of T. koksaghyz latex is slightly orange, indicating the presence of carotenoids.** Photograph of the latex pellet phase in a 1.5-mL reaction tube obtained after centrifugation.
